# Supplementary material for: Live-cell imaging of circadian clock protein dynamics in CRISPR-generated knock-in cells
Source: Nat Commun. 2021 Jun 18;12:3796. doi: 10.1038/s41467-021-24086-9 (PMC8213786; doi:10.1038/s41467-021-24086-9)
Supplement: Supplementary file 1 — Supplementary Information [file 41467_2021_24086_MOESM1_ESM.pdf]

Live-cell imaging of circadian clock protein dynamics in CRISPR-generated knock-in cells

Christian H. Gabriel[1,2], Marta del Olmo[3], Amin Zehtabian[4], Marten Jäger[5], Silke Reischl[1,2], Hannah van Dijk[1,2], Carolin Ulbricht[6,7], Asylkhan Rakhymzhan[8], Thomas Korte[9], Barbara Koller[1,2], Astrid Grudziecki[1,2], Bert Maier[1,2], Andreas Herrmann[9], Raluca Niesner[8,10], Tomasz Zemojtel[5], Helge Ewers[4], Adrián E. Granada[11], Hanspeter Herzel[3], and Achim Kramer[1,2*]

Supplementary Information

<u>Content:</u>

Supplementary Note 1: Whole exome sequencing to analyze clonal genotypes

Supplementary Note 2: Constraining the model parameters of the Relógio model to the experimental findings

Supplementary Note 3: U-2 OS cells as a model cell line to study the molecular circadian oscillator

Supplementary References

Supplementary Figures 1-10

Supplementary Tables 1-9

Supplementary Note 1: Whole exome sequencing to analyze clonal genotypes

The generation of knock-in cells using CRISPR-Cas9 and donor-plasmid mediated homology directed repair can lead to at least two sorts of unwanted side effects: Firstly, off-target activity of the Cas9 enzyme can result in *de-novo* formation of insertions or deletions (indels). Secondly, random integration of the donor plasmid can occur. Both events modify the genome of a cell, potentially altering cellular functions if they take place at functionally relevant sites (*e.g.* protein coding regions). Alterations (*i.e.* indel formation, donor-plasmid integration) in protein coding regions are more likely to be detrimental compared to alterations happening at intronic and intergenic sites, which in most cases have no or only minimal effects on gene expression and cellular function.

To evaluate, if and to what extent unwanted side-effects occurred in our knock-in cells, we used paired-end whole exome sequencing (WES) to obtain exome sequence data for six knock-in clones. These comprise four single-knock-in clones shown in Fig. 2 and Fig. 4, and the *PER2*-mScarlet-I/*CRY1*-mClover3 double knock-in clones #4 and #6 (Fig. 3 and Fig. 4) and the founding wild-type population. For each cell clone, over 99.9 % of the reads could be mapped with ~99.5% properly paired mates. Overall, more than 40 million reads per clone covered 99.2-99.3 % of the target regions, with an average coverage depth of >86 for all clones. Furthermore, we took advantage of WES data from seven wild-type clones that we had previously acquired in another project using the same sequencing platform[1].

First, we analyzed the clones' WES data for Cas9-mediated *de-novo* indel formation. After alignment to the human reference genome (hg38), between ~20,400 and 22,600 indels per clone were detected, 2-3 % of which mapped to protein coding regions, 1.3–1.4 % to ncRNA coding regions, and the remaining to intronic (69-72 %), UTR (4.8-5.2 %) and intergenic regions. From these, we filtered out all indels that were also detected in the founding wild-type population or one of the wild-type clones sequenced previously, as these indels are unlikely to be a product of Cas9 activity. This resulted in around 4,000-6,000 unique indels per clone.

To assess, which of those indels may be the result of Cas9 activity, we generated lists with potential off-target sites in the human genome by using four different prediction tools (CasOFFinder[2], allowing for up to four mismatches and RNA/DNA bulge of 1 bp, CRISPR-ML[3,4], CCtop[5] and CRISPOR[6]). For the three sgRNAs targeting *PER2*, 13,591 unique potential off-

target sites were predicted, 1,361 (10.0 %) of which map to target regions of the sequencing. For the only used sgRNA targeting *CRY1*, 4,661 unique potential off-target sites were predicted, 215(4.5 %) of which map to sequencing target regions.

Finally, we examined, whether potential Cas9 off-target sites overlap with or are in close vicinity (+/-20 bp) to our identified unique indels. As expected, we detected indels in the wild-type alleles of the on-target site, as seen by Sanger sequencing (Supplementary Fig. 2). In contrast, we did not find any Cas9-induced indel formation near the 1,576 predicted exonic off-target sites for all four sgRNAs used. Thus, the observed indels are unlikely to be the result of Cas9 off-target effects, but rather represent clonal variations or differential coverage during exome sequencing.

Secondly, we analyzed the paired-end WES data for the presence of any donor plasmid sequence in exonic regions, which would potentially disrupt a gene's function at the integration site. To this end, we remapped the WES reads to a modified reference genome that additionally contained the donor plasmid sequences. In the case of integration events, we expected to find sequencing reads that map with one end to the sequence of the donor plasmid and with the other end to that of the exon sequence, or paired reads, of which one read maps to the vector sequence and the other one to an exon.

In one clone (*CRY1*-mClover3), we observed several reads spanning a part of the *CD4* cDNA sequence in the negative selection cassette fused to a part of the 3'-homology region of *CRY1* (intron 12 of *CRY1,* Supplementary Note Fig. N1). The *CD4* parts of the sequences map exclusively to the vector as they covered several exon-junctions without intronic sequences. A possible explanation for this is a recombination of the donor vector (Supplementary Note Fig. N1a), which was then randomly integrated into a non-exonic region (integration in an exonic region would have likely resulted in further concordant read(-pairs) at the integration site). The WES likely covered the sequence, since the *CD4*-part can hybridize with the selection probes.

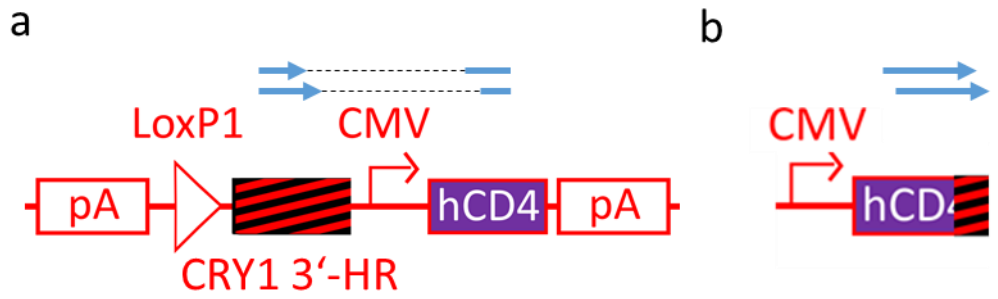

Suppl. Note Figure N1: Schematic representation of detected discordant reads (blue arrows) aligned to the donor plasmid (a) and to the rearrangement, which was detected in the genome by WES (b).

In addition, one single read in the *PER2*-mScarlet-I/*CRY1*-mClover3 double knock-in clone #4 aligned to the *CD4* coding region as well as to the *MYOF* gene on chromosome 10. We do not consider this as strong evidence for donor vector integration, because (i) the pairing of the (sub-)read to the vector region was not to an donor-specific region, *i.e.* the *CD4* coding region also exists in the genome. (ii) The genomic region, to which the other sub-read paired was well covered by >30 further reads which showed a regular pattern. In contrast, a vector integration would be expected to manifest in a more substantial proportion of reads (*e.g.* roughly 50 % for a two-allelic gene).

Furthermore, we detected four read pairs, whose one read mapped to the donor vector and the other read to an unrelated genomic region. Of note, all of the respective vector regions are also present in the genome, *i.e.* all four read pairs can also be regarded as read pairs that map to different genomic regions. With ~0.3 percent of all read pairs of the dataset (*i.e.* >100.000 per clone) pairing to different chromosomes, this is a rather common phenomenon, which can either be an artifact of the library preparation or result from genetic rearrangement. Usually, a single discordant read pair is not regarded as a strong evidence for a structural rearrangement[7]. Nonetheless, we provide the information on those reads in Supplementary Tab. 9.

In summary, we did not find evidence for *de-novo* indel formation or random integration into exonic regions. However, since WES mainly covers exonic regions, we cannot rule out the presence of the described side effects in other regions of the genome.

Supplementary Note 2: Constraining the model parameters of the Relógio model to the experimental findings

The modeling approach performed in this work was aimed to conceptualize our and others' key findings. For this reason, we took an already published model of the mammalian clockwork and modified it accordingly to fit our novel results, i.e. the higher amplitude rhythms of PER2, the delayed phase of nuclear CRY1, and the higher abundance levels of nuclear CRY1. The Relógio model is an extensive 19 variable model containing clock transcripts, cytoplasmatic and nuclear proteins, either alone or in complex with other clock proteins[8]. We reproduced the original model and translated our experimental findings to refine it (Supplementary Fig. 9), *i.e.*

(i) We simplified the PER2-CRY1 loop by removing the PER2 phosphorylation module.

(ii) We added a dissociation event of the nuclear PER2:CRY1 complex to the respective monomers.

(iii) We did not assume degradation of the cytoplasmatic and nuclear PER2:CRY1 complexes, but rather assumed degradation of the monomers after dissociation of the complex, in agreement with previous experimental work[9].

The full model equations are shown below, and the names of the variables are provided in Supplementary Tab. 6. With the aforementioned assumptions, we then systematically explored the parameter space of the PER2:CRY1 loop (Fig. 5a) by increasing and decreasing the default parameter values (published in [8], see Supplementary Tab. 7) by 250%. We prioritized parameter combinations that satisfied our experimental findings, namely (i) a circadian period, (ii) a delayed expression of nuclear CRY1 with respect to nuclear PER2, (iii) higher absolute levels of CRY1, and (iv) a larger amplitude of nuclear PER2 rhythms. Since the nuclear PER2:CRY1 dissociation event was added as a new module to the mathematical model (yellow box in Supplementary Fig. 9), we allowed association and dissociation constants, as well as PER2 and CRY1 degradation rates to be in the range of (0.01, 2.5) and (0.01, 1), respectively, values that are considered physiologically relevant[8]. With minor adjustments from the original parameter values[8] (Supplementary Tab. 7), we reproduced the experimental findings (Fig. 5b).

Nevertheless, single cells are notoriously noisy (see Fig. 2e, 5c and Supplementary Tab. 10) due to the enormous cell-to-cell variability of fundamental cellular processes, such as transcription[10], translation[11] and degradation[12]. To mimic such heterogeneity, we simulated 100 artificial cells in which we changed all transcription, translation, degradation, nuclear import and export rates (33 parameters in total). To this end, we randomly varied all corresponding 33 parameters. Values were taken from uniform distributions with means being the default parameter values (Supplementary Tab. 7) and the lower and upper limits being 90% and 110% of the default parameter value, respectively. Our simulations recapitulated the positive PER2 amplitude-period correlation observed experimentally (Fig. 5c, d).

$$(3)\,\frac{dx1}{dt} = sf(kf_{x1}x7 - kd_{x1}x1 - d_{x1}x1)$$

$$(4)\,\frac{dy3}{dt} = sf\left(V_{3max}\frac{1 + g\left(\frac{x1}{k_{t3}}\right)^{v}}{1 + \left(\frac{PC}{k_{i3}}\right)^{w}\left(\frac{x1}{k_{t3}}\right)^{v} + \left(\frac{x1}{k_{t3}}\right)^{v}} - d_{y3}y3\right)$$

$$(5)\,\frac{dy4}{dt} = sf\left(V_{4max}\frac{1 + h\left(\frac{x1}{k_{t4}}\right)^{p}}{1 + \left(\frac{PC}{k_{i4}}\right)^{q}\left(\frac{x1}{k_{t4}}\right)^{p} + \left(\frac{x1}{k_{t4}}\right)^{p}} - d_{y4}y4\right)$$

$$(6)\,\frac{dz6}{dt} = sf\big(k_{p3}(y3 + Rev_0) - ki_{z6}z6 - d_{z6}z6\big)$$

$$(7)\,\frac{dz7}{dt} = sf\big(k_{p4}(y4 + Ror_0) - ki_{z7}z7 - d_{z7}z7\big)$$

$$(8)\,\frac{dx5}{dt} = sf(ki_{z6}z6 - d_{x5}x5),$$

$$(9)\,\frac{dx6}{dt} = sf(ki_{z7}z7 - d_{x6}x6)$$

$$(10)\,\frac{dy5}{dt} = sf\left(V_{5max}\frac{1 + i\left(\frac{x6}{k_{t5}}\right)^{n}}{1 + \left(\frac{x6}{k_{t5}}\right)^{n} + \left(\frac{x5}{k_{i5}}\right)^{m}} - d_{y5}y5\right)$$

$$(11)\,\frac{dz8}{dt} = sf\big(k_{p5}(y5 + Bmal_0) - ki_{z8}z8 - d_{z8}z8\big)$$

$$(12)\,\frac{dx7}{dt} = sf(ki_{z8}z8 + kd_{x1}x1 - kf_{x1}x7 - d_{x7}x7)$$

$$(13)\,\frac{dy1}{dt} = sf\left(V_{1max}\frac{1 + a\left(\frac{x1}{k_{t1}}\right)^{b}}{1 + \left(\frac{PC}{k_{i1}}\right)^{c}\left(\frac{x1}{k_{t1}}\right)^{b} + \left(\frac{x1}{k_{t1}}\right)^{b}} - d_{y1}y1\right)$$

$$(13)\,\frac{dy1}{dt} = sf\left(V_{1max}\frac{1 + a\left(\frac{x1}{k_{t1}}\right)^{b}}{1 + \left(\frac{PC}{k_{i1}}\right)^{c}\left(\frac{x1}{k_{t1}}\right)^{b} + \left(\frac{x1}{k_{t1}}\right)^{b}} - d_{y1}y1\right)$$

$$(14)\,\frac{dy2}{dt} = sf\left(V_{2max}\frac{1 + d\left(\frac{x1}{k_{t2}}\right)^{e}}{1 + \left(\frac{PC}{k_{i2}}\right)^{f}\left(\frac{x1}{k_{t2}}\right)^{e} + \left(\frac{x1}{k_{t2}}\right)^{e}}\frac{1}{1 + \left(\frac{x5}{k_{i21}}\right)^{f1}} - d_{y2}y2\right)$$

$$(15)\,\frac{dz1}{dt} = sf\left(k_{p2}(y2 + Cry_{0}) + kd_{z5}z5 - kf_{z5}z1z2 - d_{z1}z1\right)$$

$$(16)\,\frac{dz2}{dt} = sf\left(k_{p1}(y1 + Per_{0}) + kd_{z5}z5 - kf_{z5}z1z2 - d_{z2}z2\right)$$

$$(17)\,\frac{dz5}{dt} = sf(kf_{z5}z1z2 + ke_{x3}x3 - ki_{z5}z5 - kd_{z5}z5 - d_{z5}z5)$$

$$(18)\,\frac{dx3}{dt} = sf(ki_{z5}z5 - ke_{x3}x3 - kd_{x3}x3 + kf_{x3}x8x9 - d_{x3}x3)$$

$$(19)\,\frac{dx8}{dt} = sf(kd_{x3}x3 - kf_{x3}x8x9 - d_{x8}x8)$$

$$(20)\,\frac{dx9}{dt} = sf(kd_{x3}x3 - kf_{x3}x8x9 - d_{x9}x9)$$

Supplementary Note 3: U-2 OS cells as a model cell line to study the molecular circadian oscillator

U-2 OS osteosarcoma cells are a well-accepted cellular model in the circadian field. They possess an overall intact molecular clock, *i.e.* they display robust, temperature-compensated circadian rhythmicity[13,14]. Alike murine embryonal fibroblasts (MEF) – a widely used primary cell model in the circadian field – they can be entrained or synchronized by diverse Zeitgebers, including temperature cycles and glucocorticoids, which are thought to be highly relevant in the physiological context[15,16]. On a single cell level, circadian period distribution and period inheritance shows striking similarities between U-2 OS cells and mouse fibroblasts[1,17]

Genetic manipulations of core clock genes in U-2 OS cells usually result in phenotypic changes that resemble those seen on primary MEFs. For example, *CRY1* knock-out results in a short-period phenotype, whereas *CRY2* knock-out leads to period lengthening. *CRY1/2* double knock-out or ablation of *BMAL1* results in arrhytmicity[13,18–21]. Remarkably, similar effects can

be observed on the level of SCN slices or behavior of knock-out mice, demonstrating an overall similar makeup of the molecular circadian clock over different cell types and tissues [22–25].

However, the molecular oscillators in the SCN possess some unique features that distinguish them from the 'peripheral' oscillator in other cells (reviewed in[26]). Most strikingly, extensive intracellular coupling of inherently noisy single cell circadian oscillations confers the central oscillator a superior robustness [24]. In regard of these differences, data from peripheral cells do not always reflect the SCN state.

Together, there are several reasons to assume that the fundamental molecular mechanism of the circadian clock in U-2 OS cells is very similar to that in primary cells. Indeed, the delayed CRY1 expression that we observe in U-2 OS cells is also present in mouse liver cells and fibroblasts[27,28]. Yet, we cannot exclude the possibility that genomic aberrations in these cells led to alteration in the clock protein dynamics compared to primary osteoblasts. However, the state variables of the circadian network in these cells allow for robust, free-running, entrainable and temperature compensated circadian oscillations. Thus, they represent a model of human cells that contain a functioning peripheral circadian clock (in contrast to many other transformed cell lines that are arrhythmic[29]).

Supplementary References

1. Nikhil, K. L., Korge, S. & Kramer, A. Heritable gene expression variability and stochasticity govern clonal heterogeneity in circadian period. *PLOS Biol.* **18**, e3000792 (2020).
2. Bae, S., Park, J. & Kim, J.-S. Cas-OFFinder: a fast and versatile algorithm that searches for potential off-target sites of Cas9 RNA-guided endonucleases. *Bioinformatics* **30**, 1473–1475 (2014).
3. Listgarten, J. *et al.* Prediction of off-target activities for the end-to-end design of CRISPR guide RNAs. *Nat. Biomed. Eng.* **2**, 38–47 (2018).
4. Doench, J. G. *et al.* Optimized sgRNA design to maximize activity and minimize off-target effects of CRISPR-Cas9. *Nat. Biotechnol.* **34**, 184–191 (2016).
5. Stemmer, M., Thumberger, T., del Sol Keyer, M., Wittbrodt, J. & Mateo, J. L. CCTop: An Intuitive, Flexible and Reliable CRISPR/Cas9 Target Prediction Tool. *PLoS One* **10**, e0124633 (2015).
6. Haeussler, M. *et al.* Evaluation of off-target and on-target scoring algorithms and integration into the guide RNA selection tool CRISPOR. *Genome Biol.* **17**, 148 (2016).
7. Ewing, A. D. Transposable element detection from whole genome sequence data. *Mob. DNA* **6**, 24 (2015).

8. Relógio, A. *et al.* Tuning the Mammalian Circadian Clock: Robust Synergy of Two Loops. *PLoS Comput. Biol.* **7**, e1002309 (2011).

9. Schmalen, I. *et al.* Interaction of Circadian Clock Proteins CRY1 and PER2 Is Modulated by Zinc Binding and Disulfide Bond Formation. *Cell* **157**, 1203–1215 (2014).

10. Raj, A., Peskin, C. S., Tranchina, D., Vargas, D. Y. & Tyagi, S. Stochastic mRNA Synthesis in Mammalian Cells. *PLoS Biol.* **4**, e309 (2006).

11. Sonneveld, S., Verhagen, B. M. P. & Tanenbaum, M. E. Heterogeneity in mRNA Translation. *Trends Cell Biol.* **30**, 606–618 (2020).

12. Alber, A. B., Paquet, E. R., Biserni, M., Naef, F. & Suter, D. M. Single Live Cell Monitoring of Protein Turnover Reveals Intercellular Variability and Cell-Cycle Dependence of Degradation Rates. *Mol. Cell* **71**, 1079-1091.e9 (2018).

13. Maier, B. *et al.* A large-scale functional RNAi screen reveals a role for CK2 in the mammalian circadian clock. *Genes Dev.* **23**, 708–18 (2009).

14. Beale, A. D. *et al.* Casein Kinase 1 Underlies Temperature Compensation of Circadian Rhythms in Human Red Blood Cells. *J. Biol. Rhythms* **34**, 144–153 (2019).

15. Kaneko, H., Kaitsuka, T. & Tomizawa, K. Response to Stimulations Inducing Circadian Rhythm in Human Induced Pluripotent Stem Cells. *Cells* **9**, (2020).

16. Masuda, S. *et al.* Mutation of a PER2 phosphodegron perturbs the circadian phosphoswitch. *Proc. Natl. Acad. Sci.* **117**, 10888–10896 (2020).

17. Li, Y. *et al.* Epigenetic inheritance of circadian period in clonal cells. *Elife* **9**, (2020).

18. Börding, T., Abdo, A. N., Maier, B., Gabriel, C. & Kramer, A. Generation of human CrY1 and Cry2 knockout cells using duplex CRISPR/Cas9 technology. *Front. Physiol.* **10**, 1–9 (2019).

19. Rosensweig, C. *et al.* An evolutionary hotspot defines functional differences between CRYPTOCHROMES. *Nat. Commun.* **9**, 1138 (2018).

20. Ode, K. L. *et al.* Knockout-Rescue Embryonic Stem Cell-Derived Mouse Reveals Circadian-Period Control by Quality and Quantity of CRY1. *Mol. Cell* 1–15 (2016) doi:10.1016/j.molcel.2016.11.022.

21. Xu, H. *et al.* Cryptochrome 1 regulates the circadian clock through dynamic interactions with the BMAL1 C terminus. *Nat. Struct. Mol. Biol.* **22**, 476–484 (2015).

22. Tokuda, I. T., Ono, D., Honma, S., Honma, K.-I. & Herzel, H. Coherency of circadian rhythms in the SCN is governed by the interplay of two coupling factors. *PLOS Comput. Biol.* **14**, e1006607 (2018).

23. van der Horst, G. T. *et al.* Mammalian Cry1 and Cry2 are essential for maintenance of circadian rhythms. *Nature* **398**, 627–30 (1999).

24. Liu, A. C. *et al.* Intercellular Coupling Confers Robustness against Mutations in the SCN Circadian Clock Network. *Cell* **129**, 605–616 (2007).

25. Bunger, M. K. *et al.* Mop3 Is an Essential Component of the Master Circadian Pacemaker in Mammals. *Cell* **103**, 1009–1017 (2000).

26. Mohawk, J. A., Green, C. B. & Takahashi, J. S. Central and Peripheral Circadian Clocks in Mammals. *Annu. Rev. Neurosci.* **35**, 445–462 (2012).

27. Wang, J. *et al.* Nuclear Proteomics Uncovers Diurnal Regulatory Landscapes in Mouse Liver. *Cell Metab.* **25**, 102–117 (2017).

28. Narumi, R. *et al.* Mass spectrometry-based absolute quantification reveals rhythmic variation of mouse circadian clock proteins. *Proc. Natl. Acad. Sci. U. S. A.* **113**, E3461-7 (2016).

29. Relógio, A. *et al.* Ras-Mediated Deregulation of the Circadian Clock in Cancer. *PLoS Genet.* **10**, e1004338 (2014).

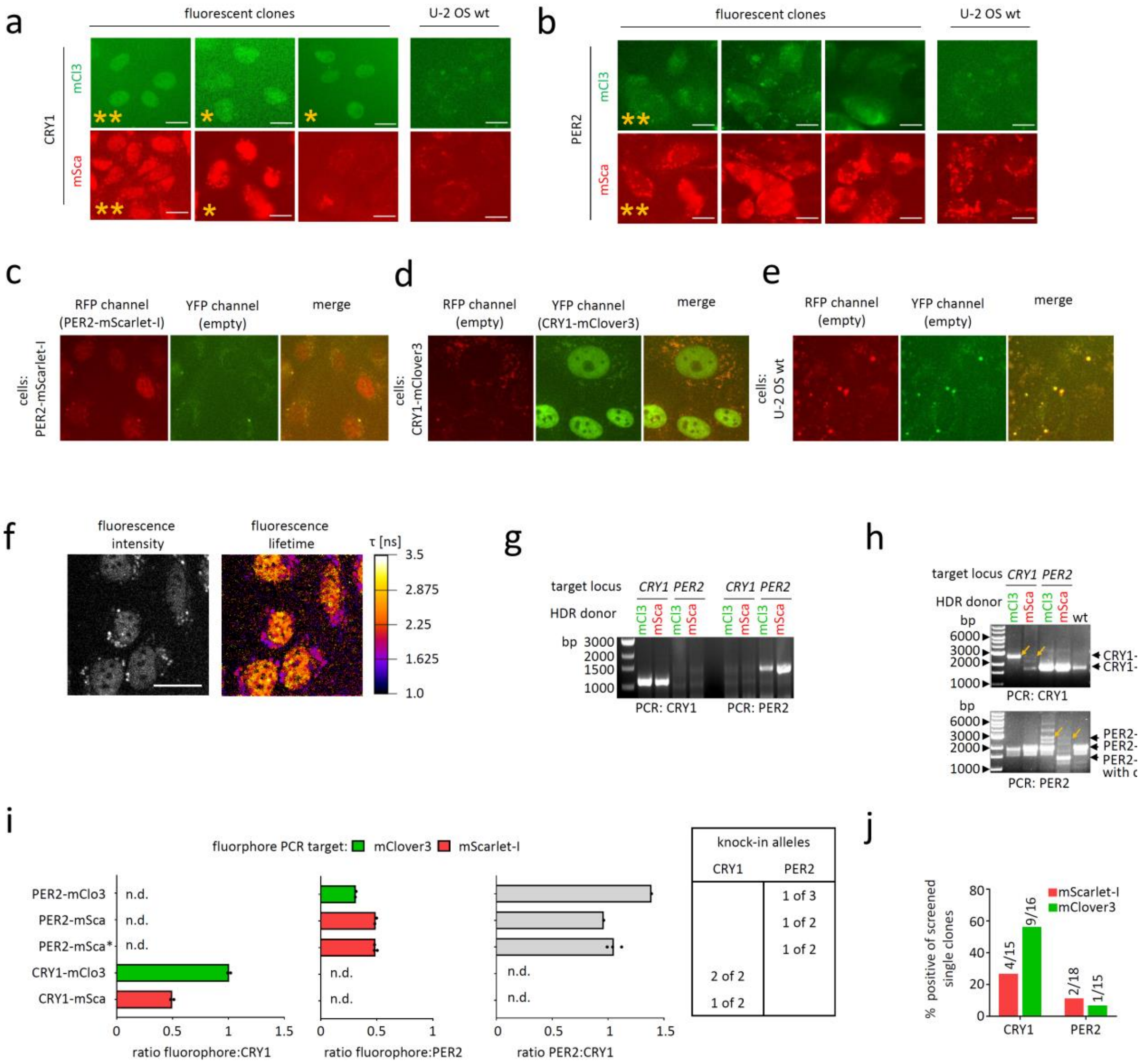

Supplementary Figure 1: Screening of potential single knock-in clones. **a** and **b**: Screening of potential CRY1 (a) and PER2 (b) knock-in clones by fluorescence microscopy using YFP and RFP channel. For each knock-in, three examples of fluorescent cells are shown along with wild-type cells that show auto-fluorescence only. Clones that were confirmed positive for correct knock-in by PCR afterwards are marked with *, clones used for all further analysis with **. **c-e**: Single knock-in (c,d) and wild-type cells (e) were imaged using YFP and RFP channel. Auto-fluorescent perinuclear signal appears in fluoresce in both channels and were also present in wild-type cells. **f** Fluorescence lifetime imaging (FLIM) of *CRY1*-mClover3 knock-in cells reveal different fluorescence lifetimes (τ) of specific nuclear and auto-fluorescent perinuclear signals. Scale bar: 30 µm. **g** Chimeric mRNA was detected in single clones by RT-PCR (as in Fig. 1c). **h** Successful knock-in was confirmed by amplification of the targeted region of

PER2 and CRY1 genomic loci by PCR, which shows either wild-type allele, the larger knock-in allele or both. Bands corresponding to knock-in alleles are marked by arrows. Additional bands are most likely heteroduplexes of wild-type and knock-in PCR product. **i** Pairwise ratios of genomic copy numbers of fluorophores and target genes were determined by ddPCR to assess the fraction of knock-in alleles. For the clone marked by (*), results obtained from the resulting double knock-in clone is shown. Bars depict the mean. **j** Percentage of positive knock-in clones in relation to all screened clones for the 4 different knock-in experiments. Numbers indicate count of correct and initially screened clonal colonies, respectively. mSca = mScarlet-I, mCl3 = mClover-3, PCR = polymerase chain reaction, n.d. = not done. HDR = homology directed repair, FP = fluorescent protein, RFP = red FP, YFP = yellow FP, wt = wild-type. Source data are provided as a Source Data file.

a

CRY1-mClover3 knock-in, schematic overview

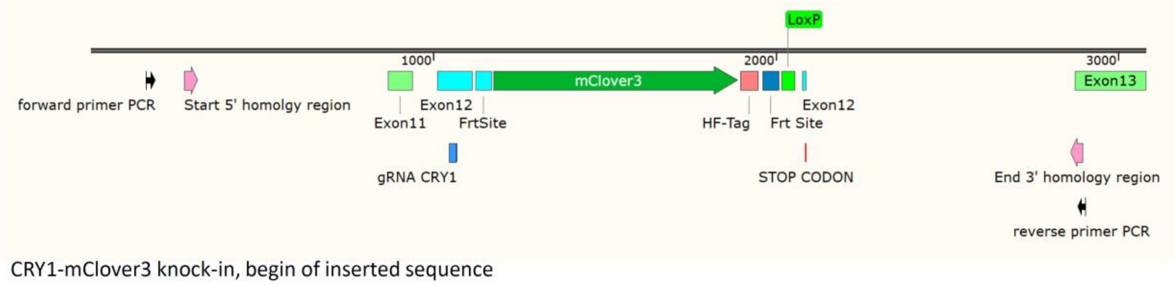

CRY1-mClover3 knock-in, begin of inserted sequence

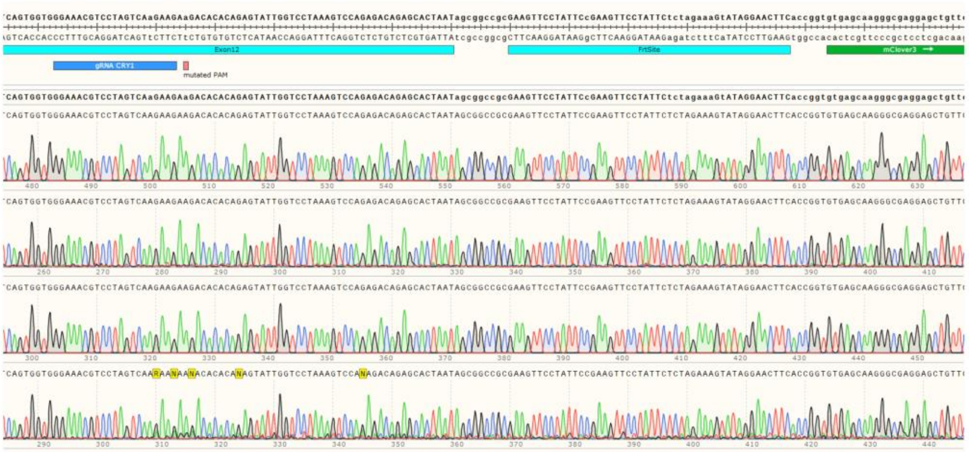

CRY1-mClover3 knock-in, end of inserted sequence

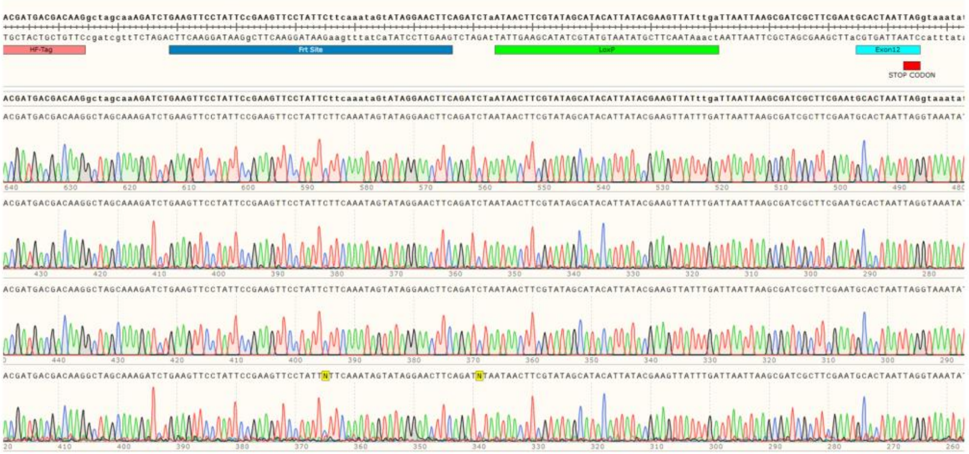

CRY1-mClover3 knock-in (wild-type allele)

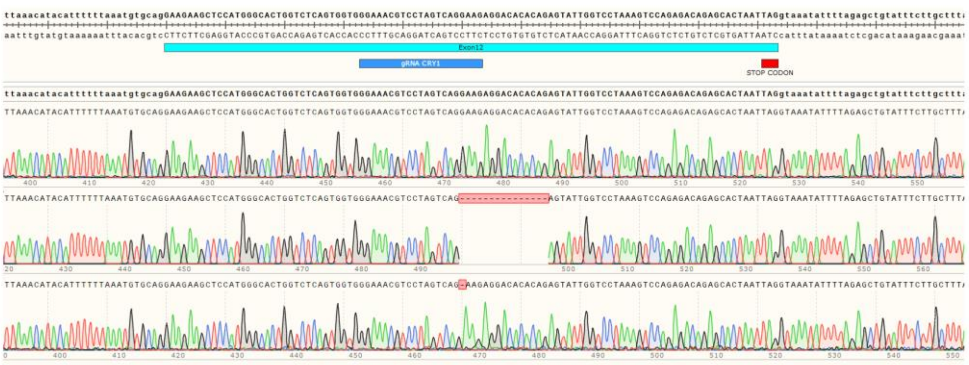

b

CRY1-mScarlet-I knock-in, schematic overview

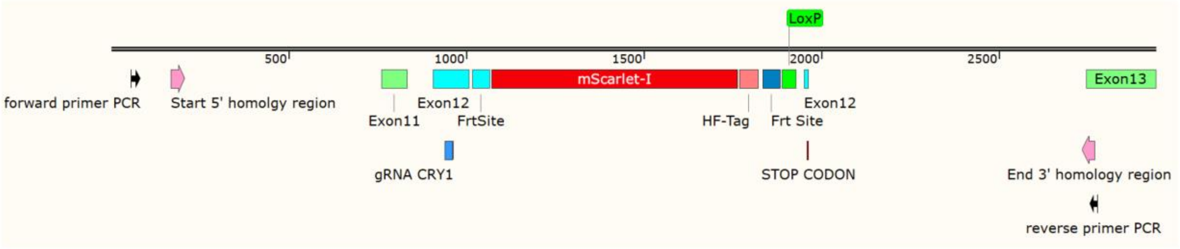

CRY1-mScarlet-I knock-in, begin of inserted sequence

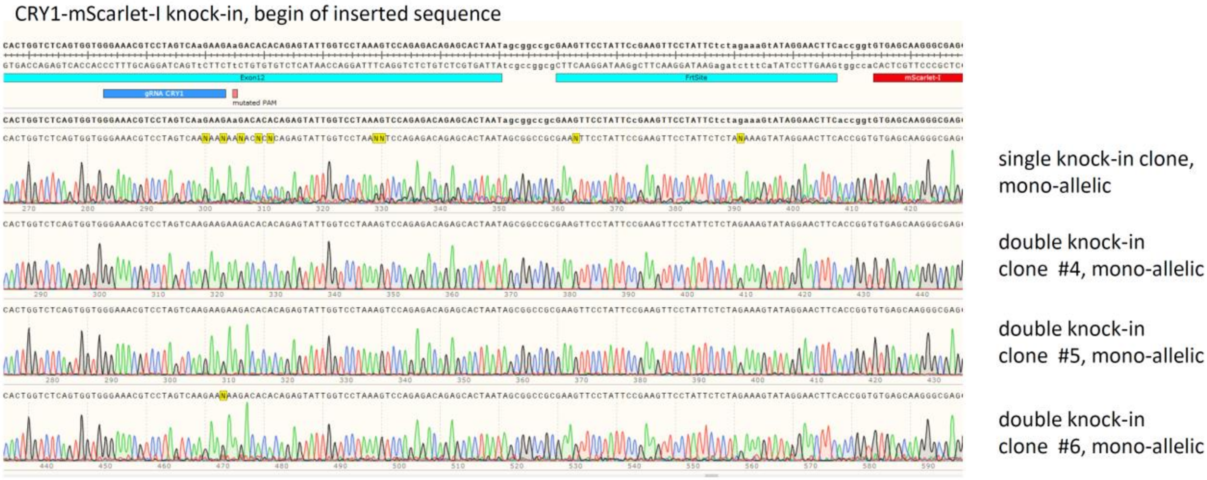

CRY1-mScarlet-I knock-in, end of inserted sequence

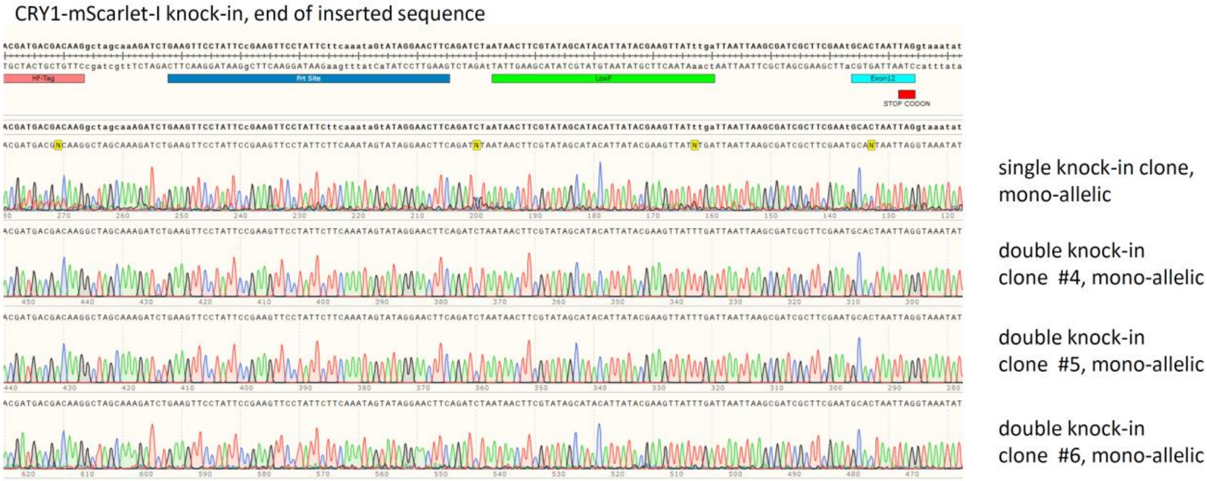

CRY1-mScarlet-I knock-in, (wild-type allele)

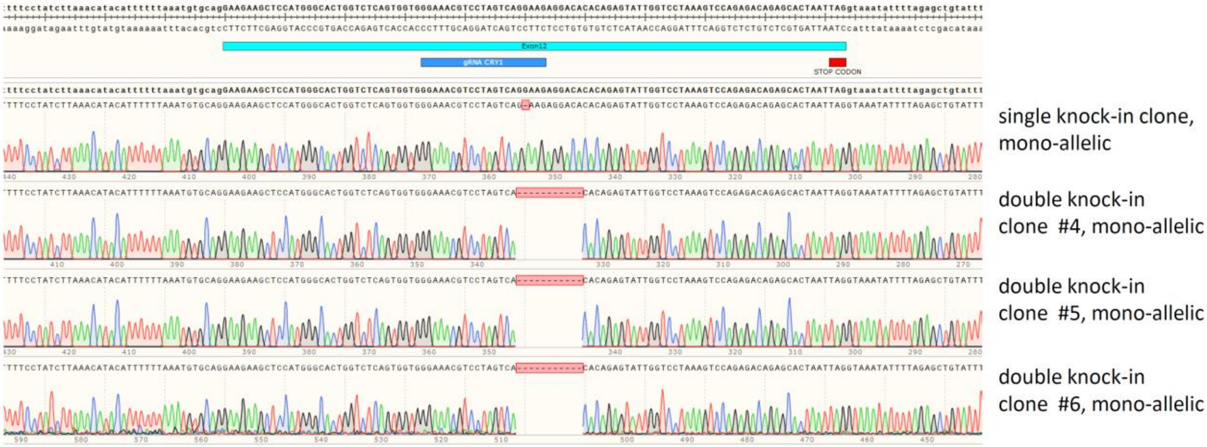

C

PER2-mClover3 knock-in, schematic overview

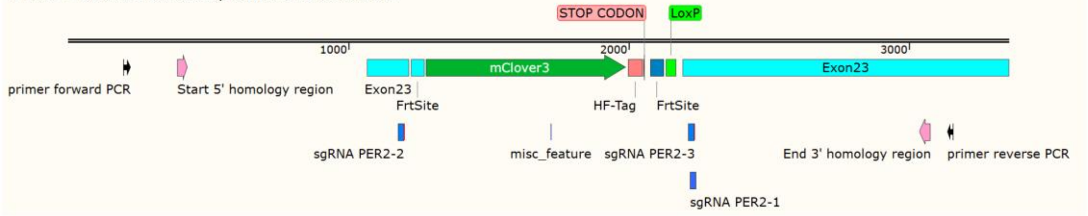

PER2-mClover3-I knock-in, begin of inserted sequence

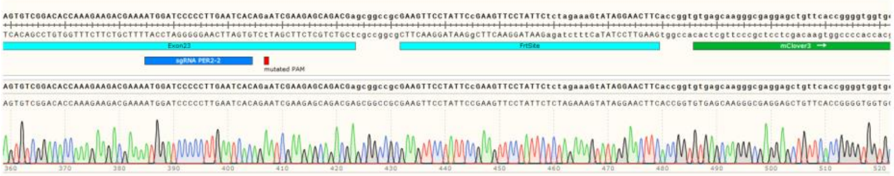

single knock-in clone
parent of double knock-in clones
mono-allelic

PER2-mClover3-I knock-in, end of inserted sequence

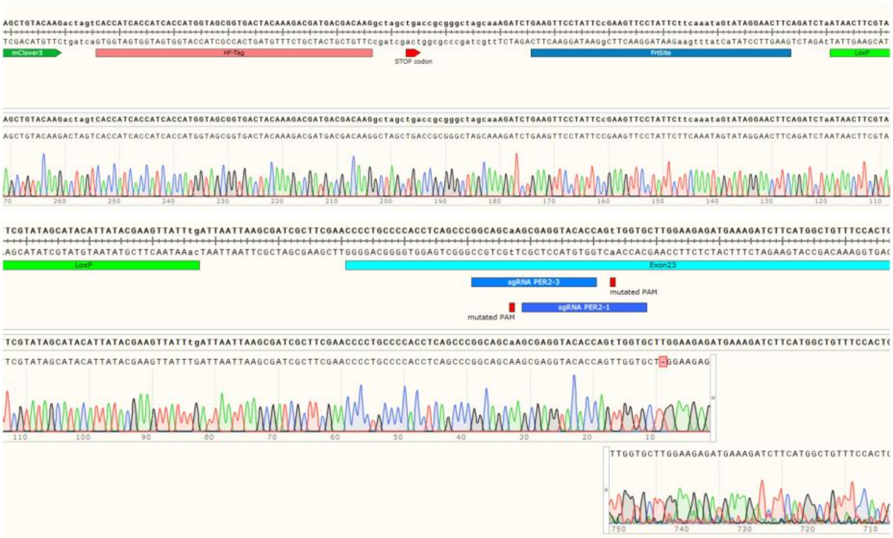

single knock-in clone
parent of double knock-in clones
mono-allelic

(continued)

PER2-mClover3-I knock-in, wild-type allele

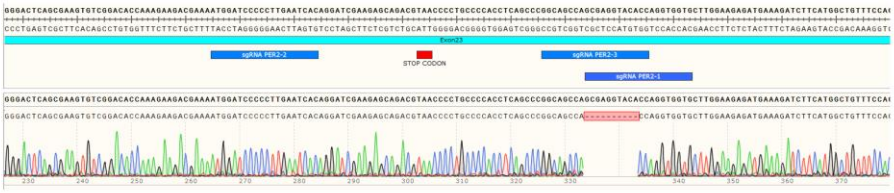

single knock-in clone
parent of double knock-in clones
mono-allelic

d

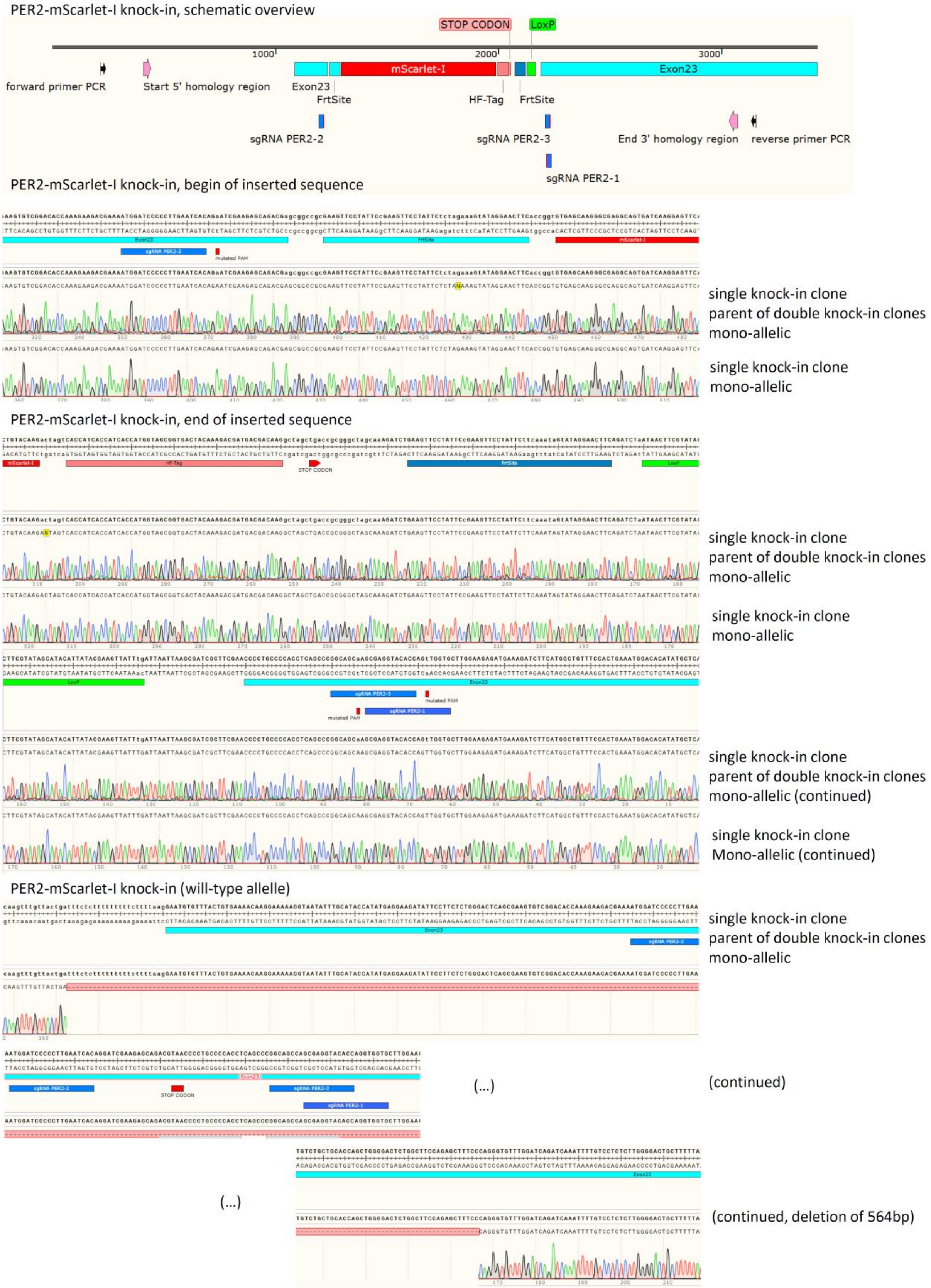

Supplementary Figure 2: Sequencing of genomic DNA of analyzed knock-in clones. Genomic DNA spanning the targeted region was PCR-amplified and analyzed by Sanger sequencing. Sequence

alignments with the expected sequences are shown for clones of *CRY1*-mClover3 (a) *CRY1*-mScarlet-I (b), *PER2*-mClover3 (c) and *PER2*-mScarlet (d) knock-in cells. Depicted are (from top to bottom): schematic view of the amplified locus, sequence spanning the beginning of the inserted sequence, sequence spanning the end of inserted sequence, wild-type loci for mono-allelic knock-in clones. For double knock-in clones, only the *CRY1* locus was sequenced, as the *PER2* locus is expected to be the same as in the parental *PER2* knock-in clones (c and d). HF-tag: His/Flag tag. PAM: protospacer adjacent motif.

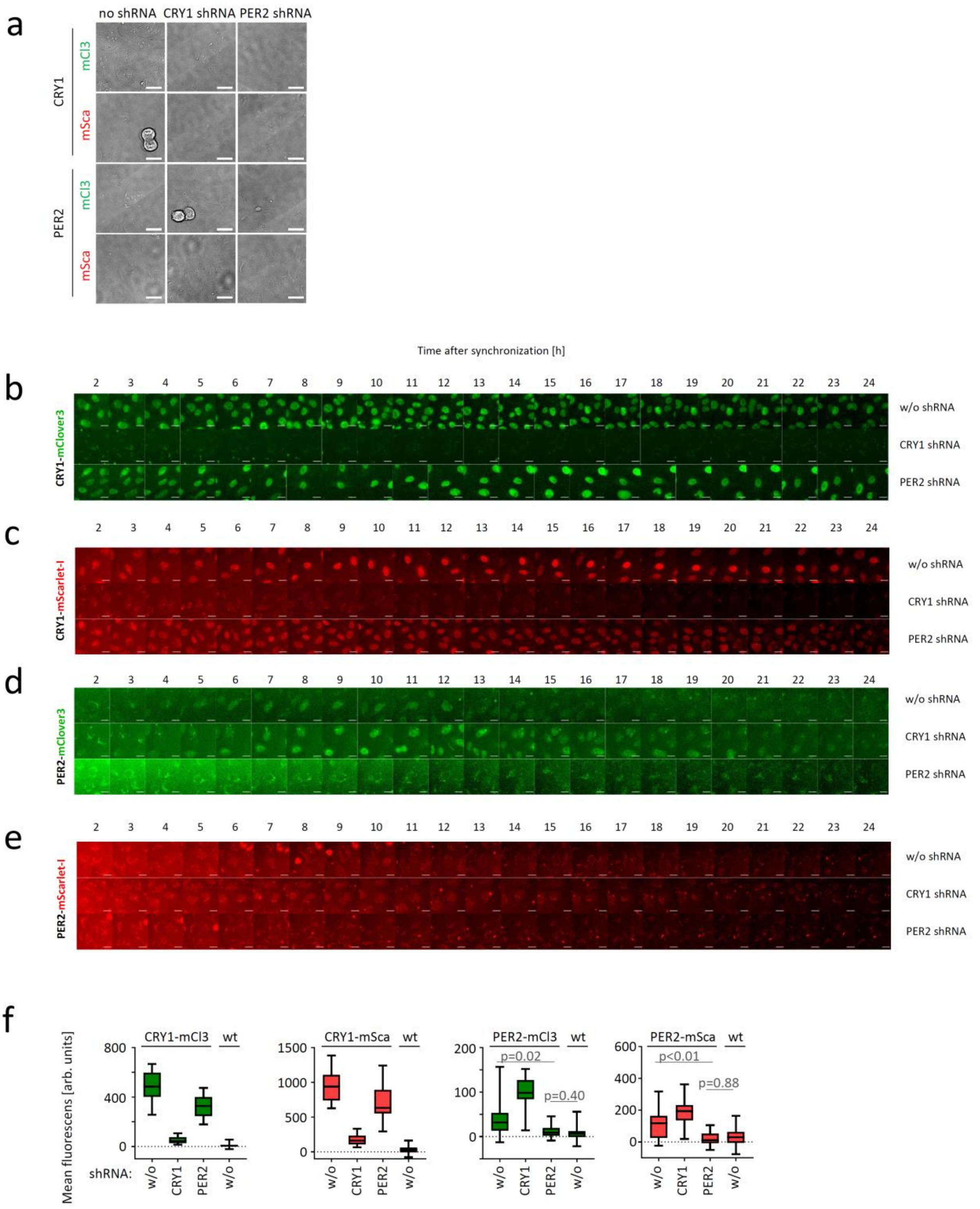

Supplemental Figure 3: Complete time series of knock-down experiment. **a** Differential interference contrast (DIC) images for Fig. 1F, scale bars: 20 µm. b-e: U-2 OS knock-in cells expressing CRY1-mClover3 (a), CRY1-mScarlet-I (c), PER2-mClover3 (d) or PER2-mScarlet-I (e), respectively, were either left untreated or transduced with shRNA targeting either *CRY1* or *PER2*. After synchronization, fluorescence in the respective channel was recorded for 24 hours. Scale bar: 20 µm. f Background subtracted mean nuclear fluorescence at 10 h after synchronization. n: 20 knock-in cells per condition,

120 non-fluorescent (wild-type) cells. Boxplots: box: interquartile range, center: median, whiskers: minimum to maximum. p-values: Kruskal-Wallis test, two-sided. mSca = mScarlet-I, mCl3 = mClover-3, shRNA = short hairpin RNA, wt = wild-type. Source data are provided as a Source Data file.

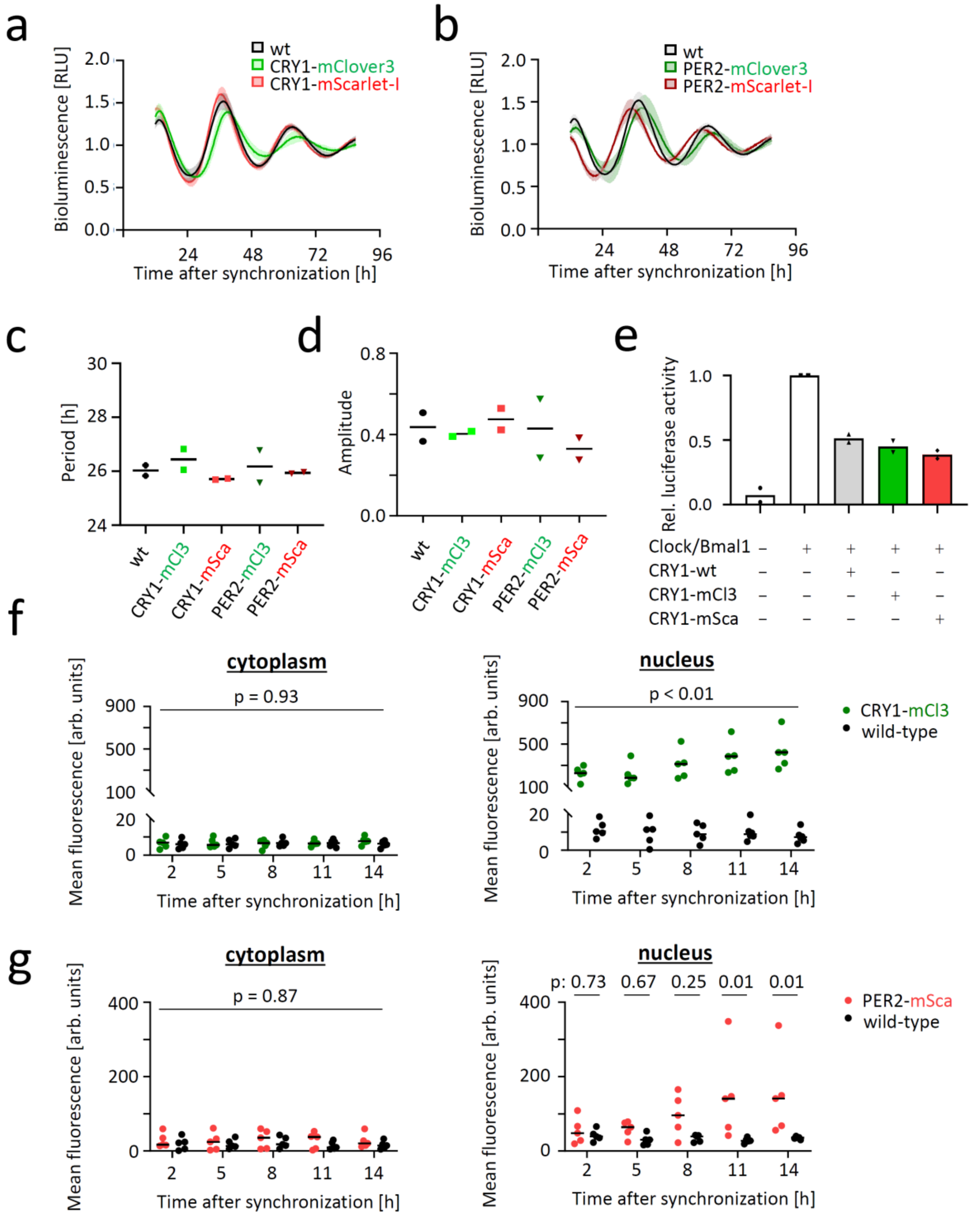

Supplementary Figure 4: Analysis of circadian rhythms and fluorescence signals in single knock-in cells. **a-d** Individual clones and wild-type cells were transduced with a *Bmal1*:Luc reporter and luminescence was recorded over four days. Depicted are mean + SD of four individual, detrended traces resulting from two independent experiments (a) and (b), and mean calculated period lengths (c) and amplitude (d) for both experiments. **e** Ability of CRY1 fusion proteins to inhibit CLOCK/BMAL1 induced activation

of an E-Box reporter plasmid. HEK-293 cells were transfected with an 6xE-Box-luciferase reporter plus the indicated constructs and reporter activity was measured (n=2 independent experiments). **f-g** Mean fluorescence signals in the cytoplasm (left) and nucleus (right) of individual *CRY1*-mClover3 (f) or *PER2*-mScarlet-I (g) knock-in cells were compared to those of wild-type cells. Data was quantified for 5 time-points spanning accumulation and peak phase of expression (n = 5 cells). p-values: one-way ANOVA, two-sided. Exact p-values for 4f, right panel (from left to right): 0.0016, 0.0016, $2.7*10^{-5}$, $9.6*10^{-7}$, $6.3*10^{-8}$. mSca = mScarlet-I, mCl3 = mClover-3, wt = wild-type. Source data are provided as a Source Data file.

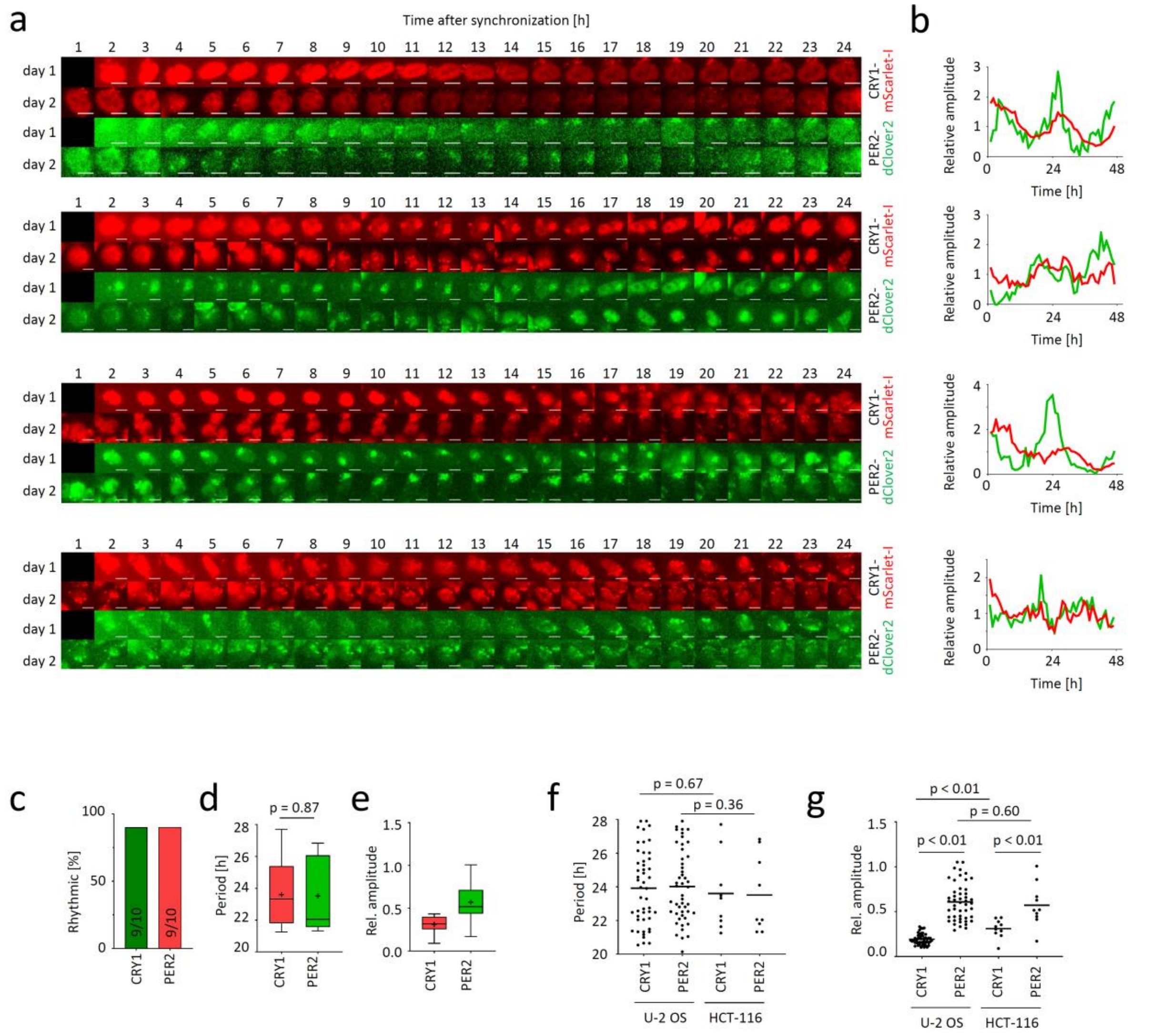

Supplementary Figure 5: Time series of HCT-116 double knock-in cells. **a** Montages of bicolor fluorescence microscopy images of individual HCT-116 double-knock-in (*PER2*-dClover2/*CRY1*-mScarlet-I) cell's nuclei over the course of 2 days. Time series of four representative individual cells are shown. Scale bar: 10 µM. **b** Mean nuclear fluorescence signals were quantified, backgrounds subtracted and signals normalized by dividing by mean signal of the time course. **c** Percentage of significantly rhythmic time series (n=10 cells). **d-e** Period and amplitude of significantly rhythmic single cell time series from (c). Boxplots: box: interquartile range, center: median, whiskers: minimum to maximum, mean is marked with (+). **f-g** Comparision of period and amplitude distribution between U-2 OS and HCT-116 double knock-in cells. p-values: Mann-Whitney-U test, two-sided (f), unpaired students's t-test, two-sided (d and g). n = 9 rythmic cells for HCT-116 and 48 rhythmic cells for U-2 OS. Bars depict mean. SKI = single knock-in, DKI = double knock-in, mSca = mScarlet-I, mCl3 = mClover3, FP = fluorescent protein, RFP = red FP, YFP = yellow FP, HFtag = 6xHis/FLAG tag, wt = wild-type. Source data are provided as a Source Data file.

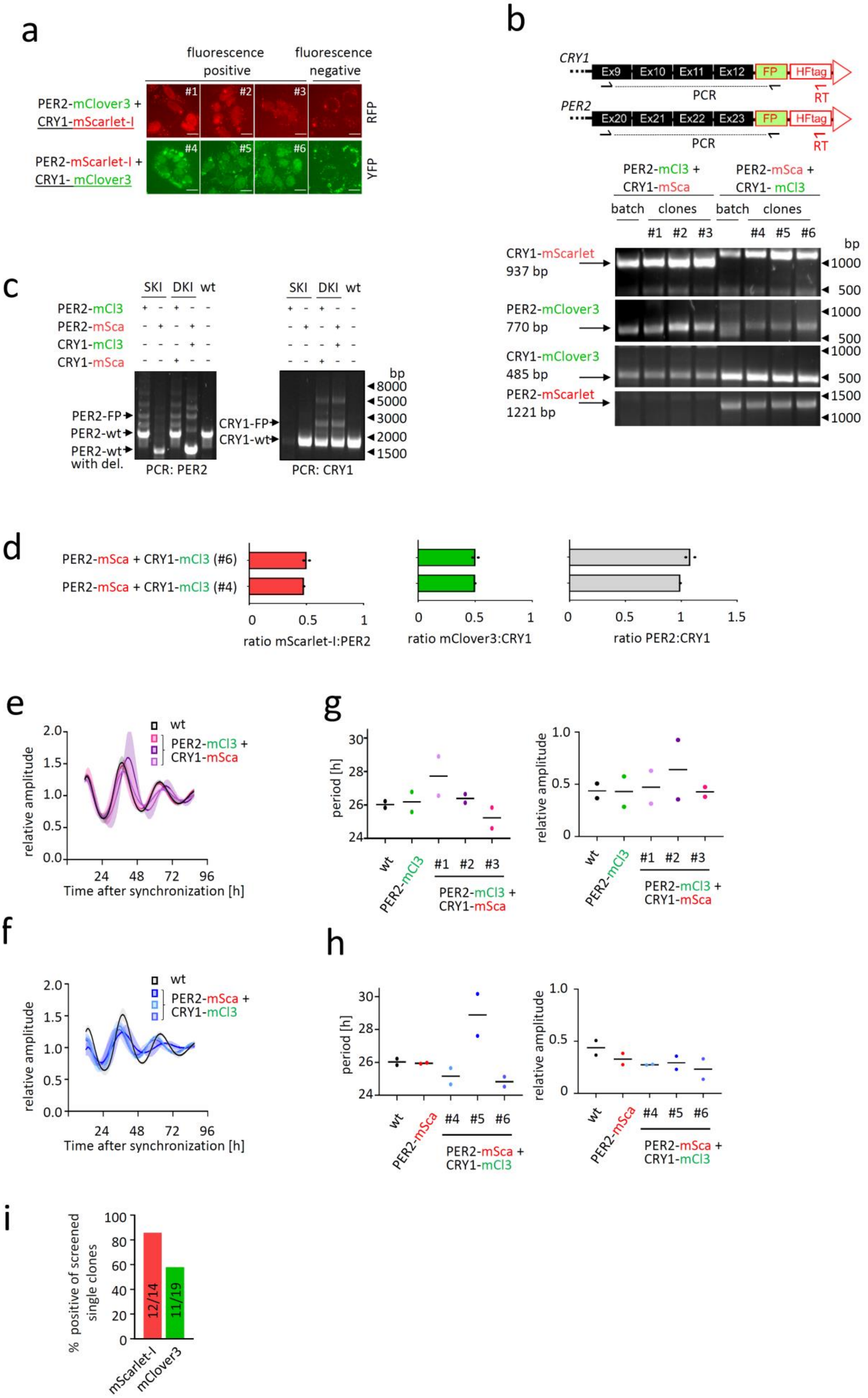
a
fluorescence positive
fluorescence negative
PER2-mClover3 +
CRY1-mScarlet-I
PER2-mScarlet-I +
CRY1- mClover3
#1
#2
#3
#4
#5
#6
RFP
YFP
b
CRY1
Ex9
Ex10
Ex11
Ex12
FP
HFtag
PCR
RT
PER2
Ex20
Ex21
Ex22
Ex23
PER2-mCl3 +
CRY1-mSca
PER2-mSca +
CRY1- mCl3
batch
clones
bp
CRY1-mScarlet
937 bp
PER2-mClover3
770 bp
CRY1-mClover3
485 bp
PER2-mScarlet
1221 bp
1000
500
1500
c
SKI
DKI
wt
PER2-mCl3
PER2-mSca
CRY1-mCl3
CRY1-mSca
PER2-FP
PER2-wt
PER2-wt with del.
PCR: PER2
CRY1-FP
CRY1-wt
PCR: CRY1
8000
5000
3000
2000
d
PER2-mSca + CRY1-mCl3 (#6)
PER2-mSca + CRY1-mCl3 (#4)
ratio mScarlet-I:PER2
ratio mClover3:CRY1
ratio PER2:CRY1
e
wt
PER2-mCl3 +
CRY1-mSca
relative amplitude
Time after synchronization [h]
f
PER2-mSca +
CRY1-mCl3
g
period [h]
PER2-mCl3
h
PER2-mSca
i
% positive of screened single clones
12/14
11/19
mScarlet-I
mClover3

Supplementary Figure 6: Selection and characterization of double knock-in clones. **a** Screening of clones with potential *CRY1*-knock in by fluorescence microscopy. For each knock-in, 3 example clones with the expected pattern are shown along with a negative clone. **b** Chimeric mRNA was detected in the three single clones from (a) by RT-PCR using RT-primer specific to the insertion, *i.e.* gene specific forward and fluorophore specific reverse primer. Arrows indicate the expected band for correct insertion. **c** Successful knock-in was confirmed by amplification of the edited genomic locus using out-out PCR followed by Sanger sequencing. Results exemplarily shown for double knock-in clones #3 and #6. **d** Pairwise ratios of genomic copy numbers of fluorophores and target genes were determined by ddPCR to assess the fraction of knock-in alleles (n = 1 - 2 independent experiments). **e-h** Individual double knock-in clones, the corresponding parental clone and wild-type cells were transduced with a *Bmal1*:luciferase reporter, and luminescence was recorded over four days. Depicted are mean ±SD of four individual, detrended time series resulting from two independent experiments (e-f), and mean period lengths and amplitudes for both experiments (n = 2 independent experiments) (g-h). Clone #6 was used for imaging analysis. i Percentage of positive knock-in clones in relation to all screened clones. Scale bar: 20 µm. mCl3 = mClover3, mSca = mScarlet-I, FP = fluorescent protein (mScarlet-I or mClover3). Source data are provided as a Source Data file.

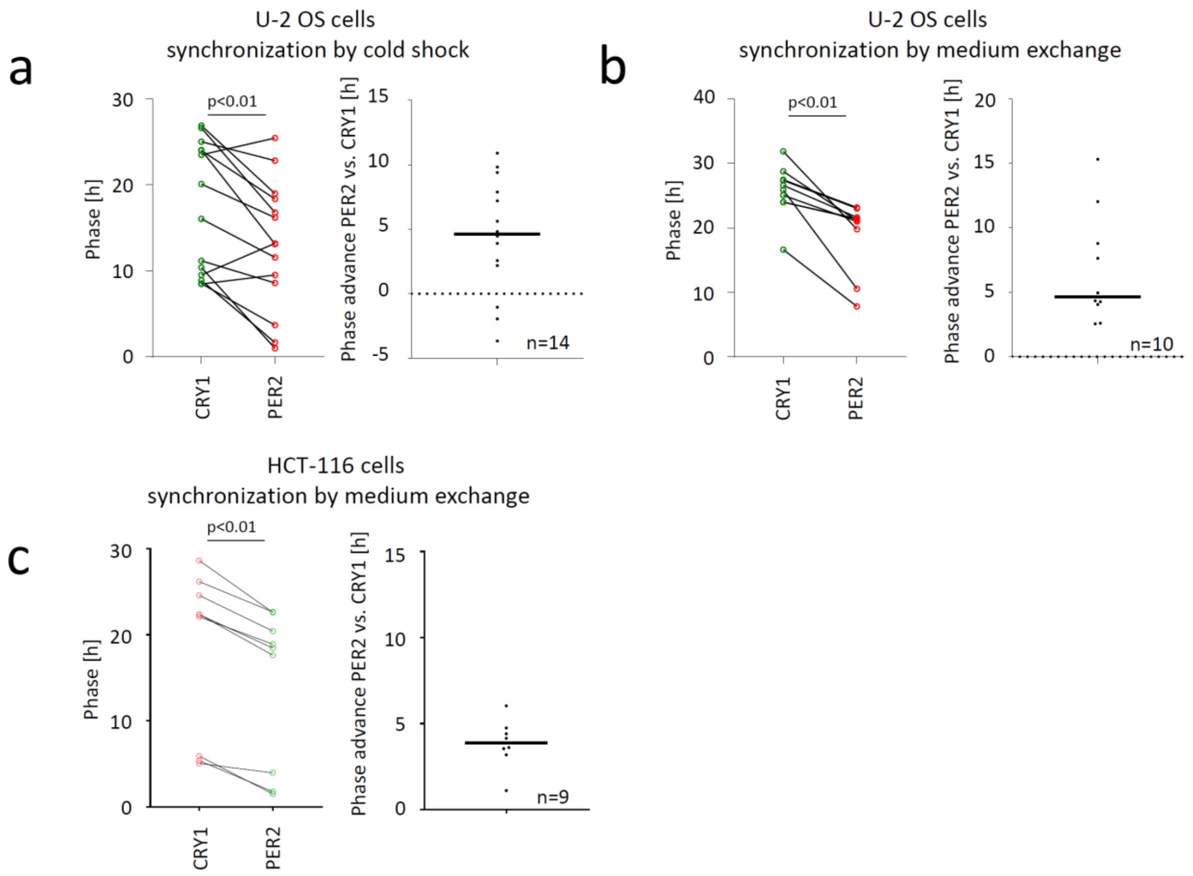

Supplementary Figure 7: Phase difference of PER2 and CRY1 under various conditions. Analysis of phase difference between CRY1 and PER2 nuclear accumulation in individual double knock in cells: **a** U-2 OS cells after synchronization by cold shock, **b** U-2 OS cells after synchronization by medium exchange, **c** HCT-116 after synchronization by medium exchange. For (a), phases were calculated excluding the first 24 hours of the time series. p-values: Wilcoxon matched-pairs signed rank test, two-sided. Numbers (n) refer to individual cells. Bars define the median. Source data are provided as a Source Data file.

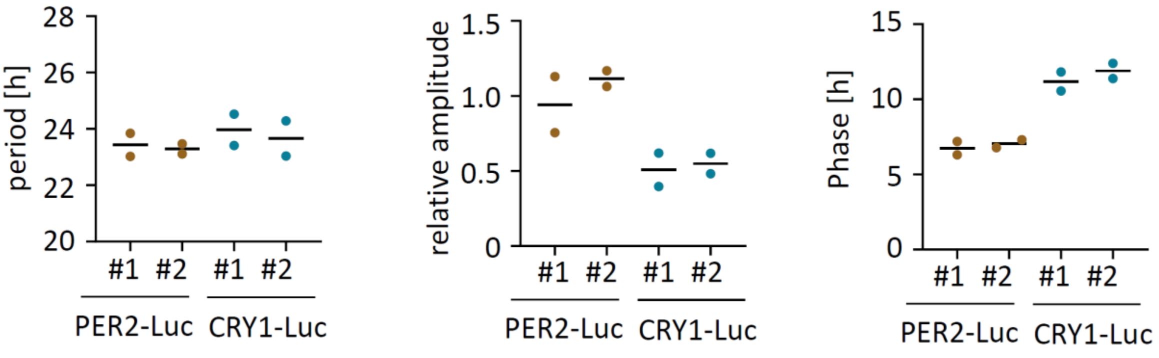

Supplementary Figure 8: Analysis of PER2-Luc and CRY1-Luc oscillations. Extracted periods, amplitudes and phases from Fig. 4C. Data from n = 2 independent experiments with two clones of each knock-in and 3 technical replicates. Bars define the median of the two experiments. Source data are provided as a Source Data file.

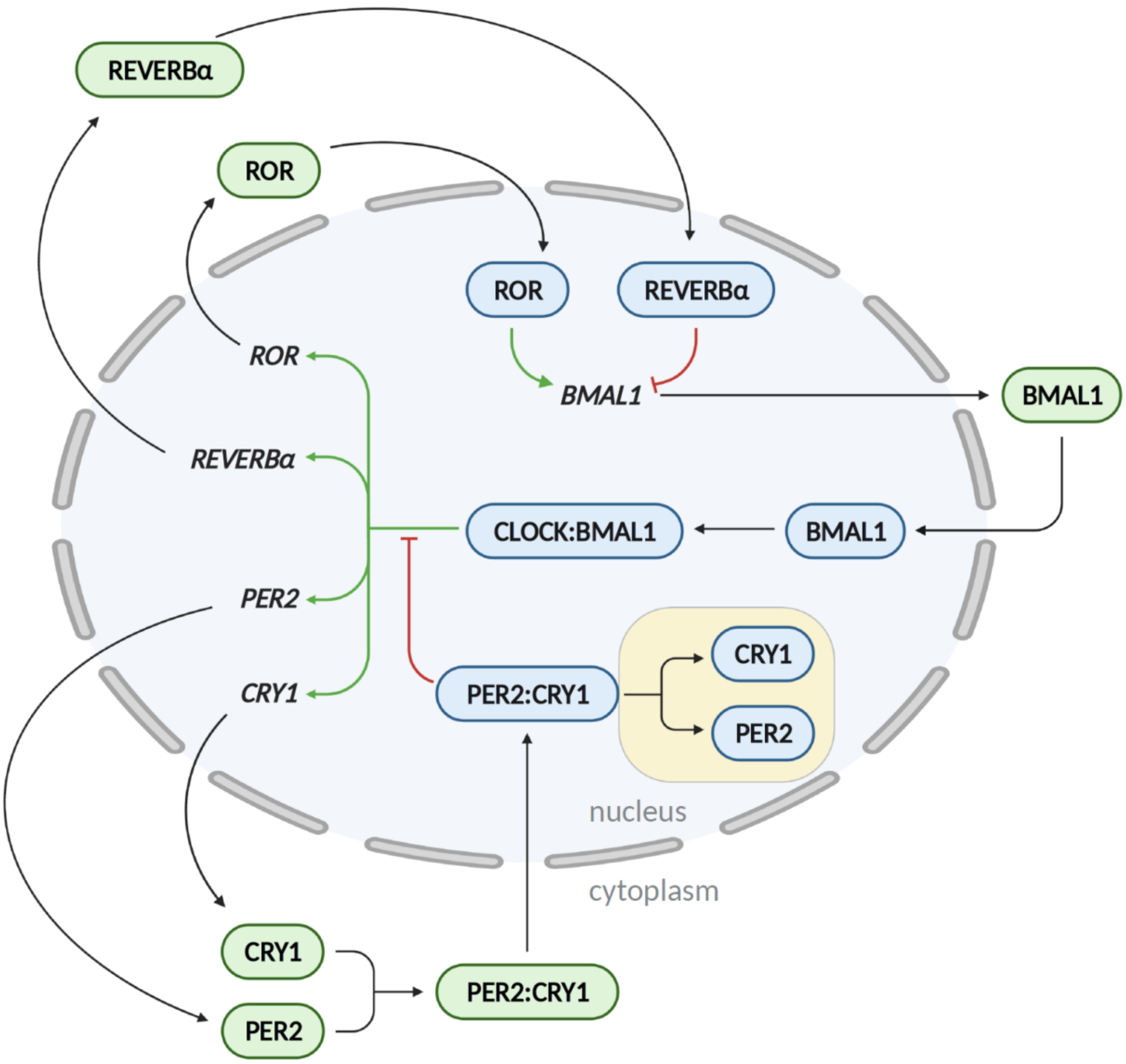

Supplementary Figure 9: Scheme of the employed conceptual model. Model was adapted from the Relógio system[8], in which nuclear dissociation of the PER2:CRY1 complex into the clock monomers is emphasized (yellow box). This additional nuclear dissociation module was added to the original model and nuclear PER2 and CRY1 monomers were modeled as explicit variables. Blue boxes indicate nuclear proteins/complexes, green boxes indicate cytoplasmatic proteins/complexes. Clock transcripts are shown in italics. Colored lines indicate positive (green) and negative (red) regulations of proteins on expression of other clock components.

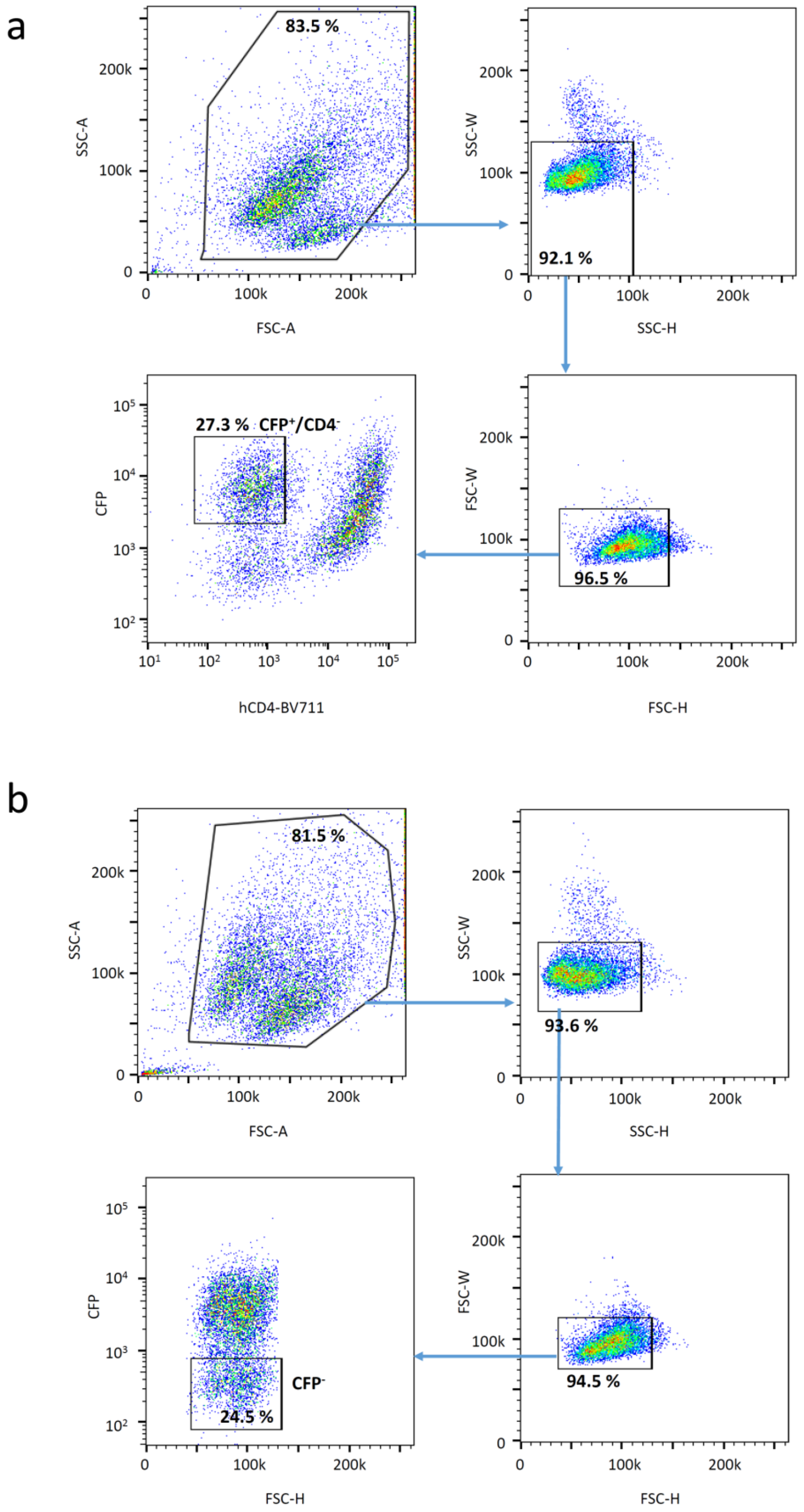

Supplementary Figure 10: Exemplary sorting strategy for positive/negative selection. Open forward scatter (FSC) and sideward scatter (SSC) were used to gate out debris and doublets before first sorting for $CD4^-$/CFP^+ cells (a) and, after CRE transfection, for CFP^- cells (b).

Supplementary Table 1: Utilized DNA sequences

Part	Sequence
His-Flag-Tag (HF-tag)	CACCATCACCATCACCATGGTAGCGGTGACTACAAAGACGATGACGACAAG
hCD4 extracellular domain	ATGAACCGGGGAGTCCCTTTTAGGCACTTGCTTCTGGTGCTGCAACTGGCGCTCC<br>TCCCAGCAGCCACTCAGGGAAAGAAAGTGGTGCTGGGCAAAAAAGGGGATACAGT<br>GGAACTGACCTGTACAGCTTCCCAGAAGAAGAGCATACAATTCCACTGGAAAAAC<br>TCCAACCAGATAAAGATTCTGGGAAATCAGGGCTCCTTCTTAACTAAAGGTCCAT<br>CCAAGCTGAATGATCGCGCTGACTCAAGAAGAAGCCTTTGGGACCAAGGAAACTT<br>CCCCCTGATCATCAAGAATCTTAAGATAGAAGACTCAGATACTTACATCTGTGAA<br>GTGGAGGACCAGAAGGAGGAGGTGCAATTGCTAGTGTTCGGATTGACTGCCAACT<br>CTGACACCCACCTGCTTCAGGGGCAGAGCCTGACCCTGACCTTGGAGAGCCCCCC<br>TGGTAGTAGCCCCTCAGTGCAATGTAGGAGTCCAAGGGGTAAAAACATACAGGGG<br>GGGAAGACCCTCTCCGTGTCTCAGCTGGAGCTCCAGGATAGTGGCACCTGGACAT<br>GCACTGTCTTGCAGAACCAGAAGAAGGTGGAGTTCAAAATAGACATCGTGGTGCT<br>AGCTTTCCAGAAGGCCTCCAGCATAGTCTATAAGAAAGAGGGGGAACAGGTGGAG<br>TTCTCCTTCCCACTCGCCTTTACAGTTGAAAAGCTGACGGGCAGTGGCGAGCTGT<br>GGTGGCAGGCGGAGAGGGCTTCCTCCTCCAAGTCTTGGATCACCTTTGACCTGAA<br>GAACAAGGAAGTGTCTGTAAAACGGGTTACCCAGGACCCTAAGCTCCAGATGGGC<br>AAGAAGCTCCCGCTCCACCTCACCCTGCCCCAGGCCTTGCCTCAGTATGCTGGCT<br>CTGGAAACCTCACCCTGGCCCTTGAAGCGAAAACAGGAAAGTTGCATCAGGAAGT<br>GAACCTGGTGGTGATGAGAGCCACTCAGCTCCAGAAAAATTTGACCTGTGAGGTG<br>TGGGGACCCACCTCCCCTAAGCTGATGCTGAGCTTGAAACTGGAGAACAAGGAGG<br>CAAAGGTCTCGAAGCGGGAGAAGGCGGTGTGGGTGCTGAACCCTGAGGCGGGGAT<br>GTGGCAGTGTCTGCTGAGTGACTCGGGACAGGTCCTGCTGGAATCCAACATCAAG<br>GTTCTGCCCACATGGTCGACCCCGGTGCAGCCAATGGCCCTGATTGTGCTGGGGG<br>GCGTCGCCGGCCTCCTGCTTTTCATTGGGCTAGGCATCTTCTTCTGTGTCAGGTG<br>CCGGCACTGA
mClover3	GTGAGCAAGGGCGAGGAGCTGTTCACCGGGGTGGTGCCCATCCTGGTCGAGCTGG<br>ACGGCGACGTAAACGGCCACAAGTTCAGCGTCCGCGGCGAGGGCGAGGGCGATGC<br>CACCAACGGCAAGCTGACCCTGAAGTTCATCTGCACCACCGGCAAGCTGCCCGTG<br>CCCTGGCCCACCCTCGTGACCACCTTCGGCTACGGCGTGGCCTGCTTCAGCCGCT<br>ACCCCGACCACATGAAGCAGCACGACTTCTTCAAGTCCGCCATGCCCGAAGGCTA<br>CGTCCAGGAGCGCACCATCTCTTTCAAGGACGACGGTACCTACAAGACCCGCGCC<br>GAGGTGAAGTTCGAGGGCGACACCCTGGTGAACCGCATCGAGCTGAAGGGCATCG

	ACTTCAAGGAGGACGGCAACATCCTGGGGCACAAGCTGGAGTACAACTTCAACAG<br>CCACTACGTCTATATCACGGCCGACAAGCAGAAGAACTGCATCAAGGCTAACTTC<br>AAGATCCGCCACAACGTTGAGGACGGCAGCGTGCAGCTCGCCGACCACTACCAGC<br>AGAACACCCCCATCGGCGACGGCCCCGTGCTGCTGCCCGACAACCACTACCTGAG<br>CCATCAGTCCAAGCTGAGCAAAGACCCCAACGAGAAGCGCGATCACATGGTCCTG<br>CTGGAGTTCGTGACCGCCGCCGGGATTACACATGGCATGGACGAGCTGTACAAG
mScarlet-I	GTGAGCAAGGGCGAGGCAGTGATCAAGGAGTTCATGCGGTTCAAGGTGCACATGG<br>AGGGCTCCATGAACGGCCACGAGTTCGAGATCGAGGGCGAGGGCGAGGGCCGCCC<br>CTACGAGGGCACCCAGACCGCCAAGCTGAAGGTGACCAAGGGTGGCCCCCTGCCC<br>TTCTCCTGGGACATCCTGTCCCCTCAGTTCATGTACGGCTCCAGGGCCTTCATCA<br>AGCACCCCGCCGACATCCCCGACTACTATAAGCAGTCCTTCCCCGAGGGCTTCAA<br>GTGGGAGCGCGTGATGAACTTCGAGGACGGCGGCGCCGTGACCGTGACCCAGGAC<br>ACCTCCCTGGAGGACGGCACCCTGATCTACAAGGTGAAGCTCCGCGGCACCAACT<br>TCCCTCCTGACGGCCCCGTAATGCAGAAGAAGACAATGGGCTGGGAAGCGTCCAC<br>CGAGCGGTTGTACCCCGAGGACGGCGTGCTGAAGGGCGACATTAAGATGGCCCTG<br>CGCCTGAAGGACGGCGGCCGCTACCTGGCGGACTTCAAGACCACCTACAAGGCCA<br>AGAAGCCCGTGCAGATGCCCGGCGCCTACAACGTCGACCGCAAGTTGGACATCAC<br>CTCCCACAACGAGGACTACACCGTGGTGGAACAGTACGAACGCTCCGAGGGCCGC<br>CACTCCACCGGCGGCATGGACGAGCTGTACAAG
CFP-P2A-BlaR	ATGGTGAGCAAGGGCGAGGAGCTGTTCACCGGGGTGGTGCCCATCCTGGTCGAGC<br>TGGACGGCGACGTAAACGGCCACAAGTTCAGCGTGTCCGGCGAGGGCGAGGGCGA<br>TGCCACCTACGGCAAGCTGACCCTGAAGTTCATCTGCACCACCGGCAAGCTGCCC<br>GTGCCCTGGCCCACCCTCGTGACCACCCTGACCTGGGGCGTGCAGTGCTTCGCCC<br>GCTACCCCGACCACATGAAGCAGCACGACTTCTTCAAGTCCGCCATGCCCGAAGG<br>CTACGTCCAGGAGCGCACCATCTTCTTCAAGGACGACGGCAACTACAAGACCCGC<br>GCCGAGGTGAAGTTCGAGGGCGACACCCTGGTGAACCGCATCGAGCTGAAGGGCA<br>TCGACTTCAAGGAGGACGGCAACATCCTGGGGCACAAGCTGGAGTACAACGCCAT<br>CAGCGACAACGTCTATATCACCGCCGACAAGCAGAAGAACGGCATCAAGGCCAAC<br>TTCAAGATCCGCCACAACATCGAGGACGGCAGCGTGCAGCTCGCCGACCACTACC<br>AGCAGAACACCCCCATCGGCGACGGCCCCGTGCTGCTGCCCGACAACCACTACCT<br>GAGCACCCAGTCCAAGCTGAGCAAAGACCCCAACGAGAAGCGCGATCACATGGTC<br>CTGCTGGAGTTCGTGACCGCCGCCGGGATCACTCTCGGCATGGACGAGCTGTACA<br>AGGAATTCGGAAGCGGAGCTACTAACTTCAGCCTGCTGAAGCAGGCTGGAGACGT<br>GGAGGAGAACCCTGGACCTCACGTGGCCAAGCCTTTGTCTCAAGAAGAATCCACC<br>CTCATTGAAAGAGCAACGGCTACAATCAACAGCATCCCCATCTCTGAAGACTACA<br>GCGTCGCCAGCGCAGCTCTCTCTAGCGACGGCCGCATCTTCACTGGTGTCAATGT<br>ATATCATTTTACTGGGGGACCTTGTGCAGAACTCGTGGTGCTGGGCACTGCTGCT<br>GCTGCGGCAGCTGGCAACCTGACTTGTATCGTCGCGATCGGAAATGAGAACAGGG

	GCATCTTGAGCCCCTGCGGACGGTGCCGACAGGTGCTTCTCGATCTGCATCCTGG<br>GATCAAAGCCATAGTGAAGGACAGTGATGGACAGCCGACGGCAGTTGGGATTCGT<br>GAATTGCTGCCCTCTGGTTATGTGTGGGAGGGCTAA
LoxP Site	ATAACTTCGTATAGCATACATTATACGAAGTTAT
FrtF Site	GAAGTTCCTATTCcGAAGTTCCTATTCtctagaaaGtATAGGAACTTC
Frt3 Site	GAAGTTCCTATTCcGAAGTTCCTATTCttcaaataGtATAGGAACTTC
dClover2	GTGAGCAAGGGCGAGGAGCTGTTCACCGGGGTGGTGCCCATCCTGGTCGAGCTGG<br>ACGGCGACGTAAACGGCCACAAGTTCAGCGTCCGCGGCGAGGGCGAGGGCGATGC<br>CACCATCGGCAAGCTGACCCTGAAGTTCATCTGCACCACCGGCAAGCTGCCCGTG<br>CCCTGGCCCACCCTCGTGACCACCTTCGGCTACGGCGTGGCCTGCTTCAGCCGCT<br>ACCCCGACCACATGAAGCAGCACGACTTCTTCAAGTCCGCCATGCCCGAAGGCTA<br>CGTCCAGGAGCGCACCATCTACTTCAAGGACGACGGTACCTACAAGACCCGCGCC<br>GAGGTGAAGTTCGAGGGCGACACCCTGGTGAACCGCATCGAGCTGAAGGGCATCG<br>ACTTCAAGGAGGACGGCAACATCCTGGGGCACAAGCTGGAGTACAACTTCAACAG<br>CCACTACGTCTATATCACGGCCGACAAGCAGAACAACAGCATCAAGGCTAACTTC<br>ACCATCCGCCACAACGTTGAGGACGGCAGCGTGCAGCTCGCCGACCACTACCAGC<br>AGAACACCCCCATCGGCGACGGCCCCGTGCTGCTGCCCGACAACCACTACCTGAG<br>CCATCAGTCCGCCCTGAGCAAAGACCCCAACGAGAAGCGCGATCACATGGTCCTG<br>CTGGAGTTCGTGACCGCCGCCGGGATTACACATGGCATGGACGAGCTGTACAAG

Supplementary Table 2: Single guide RNAs sequences

Target	Guide sequence	Target sequence fw strand (PAM underlined)
CRY1 (fw strand)	GAAACGTCCTAGTCAGGAAG	GAAACGTCCTAGTCAGGAAGAGG
PER2-1 (rv strand)	CACCACCTGGTGTACCTCGC	CCAGCGAGGTACACCAGGTGGTG
PER2-2 (fw strand)	ATGGATCCCCCTTGAATCAC	ATGGATCCCCCTTGAATCACAGG
PER2-3 (fw strand)	GGCAGCCAGCGAGGTACACC	GGCAGCCAGCGAGGTACACCAGG

Supplementary Table 3: shRNA contruct sequences

Target	Hairpin sequence (targeting sequence in capital letters)
PER2 (pGIPZ V2LHS_52938)	tgctg ttgac agtga gcgcg CATCC ATATT TCACT GTAAa tagtg aagcc acaga tgtat ttaca gtgaa atatg gatgc atgcc tactg cctcg ga
CRY1 (pGIPZ V2LHS_172866)	tgctg ttgac agtga gcgcg CTGAG GCAAG CCGTT TGAAt tagtg aagcc acaga tgtaa ttcaa acggc ttgcc tcagc atgcc tactg cctcg ga

Supplementary Table 4: PCR primer sequences

Target	Sequence	Usage	Figure
CRY1 genomic locus (fw)	ACTGCCACTGATTGCCTGGGATTGAAG	fw Primer genomic PCR	SF 1h, SF 6c
CRY1 genomic locus (rv)	CAGCTGCAACAGTATTCCTCCTG	Rv primer Genomic PCR	SF 1h, SF 6c
CRY1 genomic locus (fw)	GCAAAGTAATTTGTTCTCCCAG	ddPCR	SF 1i, SF 6d
CRY1 genomic locus (rv)	TTCCAAACGAGTAAGTGCTT	ddPCR	SF 1i, SF 6d
PER2 genomic locus (fw)	ACCGGCTTCCAGGAGCCTCACTTGCA	fw Primer genomic PCR	SF 1h, SF 6c
PER2 genomic locus (rv)	AAGCTGTCAGACTGAGTGGC	Rv primer Genomic PCR	SF 1h, SF 6c
PER2 genomic locus (fw)	AGCTCCCAGAAACAACAAGG	ddPCR	SF 1i, SF 6d
PER2 genomic locus (rv)	CTTATCCTGGCCACACTGC	ddPCR	SF 1i, SF 6d
HF tag (rv)	TTGCTAGCCTTGTCGTCATC	RT Primer	1c, SF 1g, SF 6b
HF tag (rv)	ATCGTCTTTGTAGTCACCGCTACC	Rv primer RT-PCR	1c, SF 1g
CRY1 mRNA	TGCTGAGGCAAGCCGTTTGA	Fw primer RT-PCR	1c, SF 1g
PER2 mRNA	ACGCCCTTTCCACGTCAAGC	Fw primer RT-PCR	1c, SF 1g
mClover3	ACGCTGAACTTGTGGCCGTTT	Rv primer RT-PCR	SF 6b
mClover3 (fw)	GGAGCGCACCATCTCTTTCA	ddPCR	SF 1i, SF 6d
mClover3 (rv)	TGAAGTCGATGCCCTTCAGC	ddPCR	SF 1i, SF 6d
mScarlet-I	GTCTTGAAGTCCGCCAGGTAGC	Rv primer	SF 6b

		RT-PCR	
mScarlet-I (fw)	GATCTACAAGGTGAAGCTCC	ddPCR	SF 1i, SF 6d
mScarlet-I (fw)	CCTTGTAGGTGGTCTTGAAG	ddPCR	SF 1i, SF 6d

Supplementary Table 5: Sequences of TaqMen Probes

Target	Sequence	Concentration used
CRY1	HEX – TCCTCTGCAGTGTGGCCAGGTGGAGA – BHQ1	250 nM
PER2	HEX – CTGCCAGGCTAGTACAGGGTGGCCC – BHQ1	136 nM
mClover3	FAM – ACCCGCGCCGAGGTGAAGTTCGA – BHQ1	136 nM
mScarlet-I	FAM – TGGGCTGGGAAGCGTCCACCGA – BHQ1	250 nM

Supplementary Table 6: Model variables

Variable [a.u.]	Name
x1	CLOCK/BMAL1
x3	$PER2_N/CRY1_N$
PC	$PER2/CRY1_{pool}$
x5	$REVERB_N$
x6	ROR_N
x7	$BMAL1_N$
x8	$PER2_N$
x9	$CRY1_N$
y1	*PER2*
y2	*CRY1*
y3	*REVERBα*
y4	*ROR*
y5	*BMAL1*
z1	$CRY1_C$
z2	$PER2_C$
z5	$PER2_C/CRY1_C$
z6	$REVERB_C$
z7	ROR_C
z8	$BMAL1_C$

Supplementary Table 7: Parameter set of the refined Relógio model. Default parameters were adjusted to fit our experimental results. New parameters introduced in the system to model PER2:CRY1 nuclear dissociation are marked in by ‡. Parameter that were changed compared to the original model are marked by * (default parameter values in brackets).

Parameters	Name	Value
	Degradation rates for nuclear proteins or nuclear protein complexes [hour^{-1}]	
d_{x1}	CLOCK/BMAL1	0.08
d_{x3}	$PER2_N/CRY1_N$*	0 (0.09)
d_{x5}	$REVERB_N$	0.17
d_{x6}	ROR_N	0.12
d_{x7}	$BMAL1_N$	0.15
d_{x8}	$PER2_N$‡	0.3
d_{x9}	$CRY1_N$‡	0.05
	Degradation rates for mRNAs [hour^{-1}]	
d_{y1}	*PER2*	0.3
d_{y2}	*CRY1*	0.2
d_{y3}	*REVERB*	2
d_{y4}	*ROR*	0.2
d_{y5}	*BMAL1*	1.6
	Degradation rates for cytoplasmic proteins [hour^{-1}]	
d_{z1}	$CRY1_C$	0.23
d_{z2}	$PER2_C$	0.25
d_{z5}	$PER2_C/CRY1_C$*	0 (0.2)
d_{z6}	$REVERB_C$	0.31
d_{z7}	ROR_C	0.3
d_{z8}	$BMAL1_C$	0.73
	Reaction rates for complex formation/dissociation	
kf_{x1}	CLOCK/BMAL1-complex formation [hour^{-1}]	2.3
kd_{x1}	CLOCK/BMAL1-complex dissociation [hour^{-1}]	0.01
kf_{z5}	$PER2_C/CRY1_C$-complex formation [(a.u. hour)$^{-1}$] *	2.5 (1)
kd_{z5}	$PER2_C/CRY1_C$-complex dissociation [hour^{-1}] *	0.5 (1)
kf_{x3}	$PER2_N/CRY1_N$-complex formation [(a.u. hour)$^{-1}$] ‡	0.1
kd_{x3}	$PER2_N/CRY1_N$-complex dissociation [hour^{-1}] ‡	2.5
	Transcription rates [a.u. hour^{-1}]	
V_{1max}	*PER2*	1

V_{2max}	*CRY1*	2.92
V_{3max}	*REVERBα*	1.9
V_{4max}	*ROR*	10.9
V_{5max}	*BMAL1*	1
	Activation/inhibition rates [a.u.]	
k_{t1}	*PER2*-activation rate	3
k_{i1}	*PER2*-inhibition rate	0.9
k_{t2}	*CRY1*-activation rate	2.4
k_{i2}	*CRY1*-inhibition rate	0.7
k_{i21}	*CRY1*-inhibition rate	5.2
k_{t3}	*REVERB*-activation rate	2.07
k_{i3}	*REVERB*-inhibition rate	3.3
k_{t4}	*ROR*-activation rate	0.9
k_{i4}	*ROR*-inhibition rate	0.4
k_{t5}	*BMAL1*-activation rate	8.35
k_{i5}	*BMAL1*-inhibition rate	1.94
	Transcription fold activation (dimensionless)	
a	*PER2*	12
d	*CRY1*	12
g	*REVERBα*	5
h	*ROR*	5
i	*BMAL1*	12
	Production rates [hour^{-1}]	
k_{p1}	$PER2_C$	0.4
k_{p2}	$CRY1_C$	0.26
k_{p3}	$REVERBα_C$	0.37
k_{p4}	ROR_C	0.76
k_{p5}	$BMAL1_C$	1.21
	Import/Export rates [hour^{-1}]	
ki_{z5}	$PER2_C/CRY1_C$	0.1
ki_{z6}	$REVERB_C$	0.5
ki_{z7}	ROR_C	0.1
ki_{z8}	$BMAL1_C$	0.1
ke_{x3}	$PER2_N/CRY1_N$	0.01
	Hill coefficients of transcription (dimensionless)	
b	*PER2*-activation	5
c	*PER2*-inhibition	7
e	*CRY1*-activation rate	6

f	*CRY1*-inhibition	4
f1	*CRY1*-inhibition	1
v	*REVERB*-activation	6
w	*REVERB*-inhibition	2
p	*ROR*-activation	6
q	*ROR*-inhibition	3
n	*BMAL1*-activation	2
m	*BMAL1*-inhibition	5
	Exogenous RNA [a.u.]	
Per_0	*PER2*	0
Cry_0	*CRY1*	0
Rev_0	*REVERB*	0
Ror_0	*ROR*	0
$Bmal_0$	*BMAL1*	0
	Scaling factors [a.u.]	
a3		1
sf		1.07

Supplementary Table 8: Test statistics for null hypothesis testing

Figure	Applied test	Multiple comparision test	p-value (two-tailed)	Effect size	t-value	Degree of freedom	F-value	CI (95 %)
2g	ANOVA	Tukey	0,2361			78	1.4	
2i (mClover3)	ANOVA	Tukey	0,0367	3,088		78	26.9	0,18-6,04
2i (mScalet-I)	ANOVA	Tukey	0,0111	3,533		78	26.9	0,62-6,45
3f	Mann-Whitney-U		0,7830	0,1956				
3h	Mann-Whitney-U		<0,0001	5,154				
4a	Wilcoxon signed pair rank test		<0,0001	5,426				
4b	Wilcoxon signed pair rank test		<0,0001	4,911				
4f	Mann-Whitney-U		<0,0001	2,177				
4g	Mann-Whitney-U		<0,0001	2,575				
4i	Mann-Whitney-U		<0,0001	1,500				
SF 3f (PER2-mClover vs untreated)	Kruskal-Wallis-Test	Dunns	0,0297	80,15				
SF 3f (PER2-mClover vs wt)	Kruskal-Wallis-Test	Dunns	0,3990	31,08				
SF 3f (PER2-mScarlet vs untreated)	Kruskal-Wallis-Test	Dunns	0,0020	108,5				
SF 3f (PER2-mScarlet vs wt)	Kruskal-Wallis-Test	Dunns	0,8859	18,58				
SF 4f (left)	ANOVA		0,9354			42	0.4	
SF 4f(right) (ZT2)	ANOVA	Holm-Sidak's	0,0016	213,2	3,6	40	16.7	
SF 4f(right) (ZT5)	ANOVA	Holm-Sidak's	0,0016	208,9	3,5	40	16.7	
SF 4f(right) (ZT8)	ANOVA	Holm-Sidak's	<0.0001	299,3	5,1	40	16.7	
SF 4f(right) (ZT11)	ANOVA	Holm-Sidak's	<0.0001	365,7	6,2	40	16.7	
SF 4f(right)	ANOVA	Holm-Sidak's	<0.0001	419,6	7,1	40	16.7	

(ZT14)								
SF 4g(left)	ANOVA		0,8737			40	0.5	
SF 4g(right) (ZT2)	ANOVA	Holm-Sidak's	0,7331	12,45	0,76	40	3.4	
SF 4g(right) (ZT5)	ANOVA	Holm-Sidak's	0,6711	29,13	0,54	40	3.4	
SF 4g(right) (ZT8)	ANOVA	Holm-Sidak's	0,2572	62,12	0,75	40	3.4	
SF 4g(right) (ZT11)	ANOVA	Holm-Sidak's	0,0099	120,0	1,12	40	3.4	
SF 4g(right) (ZT14)	ANOVA	Holm-Sidak's	0,0114	115,2	0,93	40	3.4	
SF 5d	T-Test (unpaired		0,9339	0,09	0,08	16		-2,4-2,2
SF 5f (CRY1)	Mann-Whitney-U		0,6725	0,61				
SF 5f(PER2)	Mann-Whitney-U		0,3671	1,69				
SF 5g(CRY1)	T-Test (unpaired)		<0.0001	0.1193	4.95	56		0.07-0.17
SF 5g(PER2)	T-Test (unpaired)		0.5960	0.0395	0.53	56		-0.19-0.11
SF 5g(U-2 OS)	T-Test (unpaired)		<0.0001	0.4182	13.35	94		0.35-0-48
SF 5g(HCT-116)	T-Test (unpaired)		0,0051	0,2594	3.192	18		0,09-0,43
SF 7a	Wilcoxon signed pair rank test		0,0031	4,648				
SF 7b	Wilcoxon signed pair rank test		0,0020	4,653				
SF 7c	Wilcoxon signed pair rank test		0,0039	3,885				

Supplementary Table 9: Read pairs that align to donor vector and genomic region

Clone	Read region in donor vector	Read position in genome	Gene	Frequency / total reads at position
PER2-mClover3	*PER2* intron 23 (3'-homology region)	Chr3: 113301196	CFAP44 (intron)	1/1
PER2-mClover3	*CD4*	Chr16: 82080438	HSD17B2 (intron)	1/1
CRY1-mScarlet-I	*CD4*	Chr7: 28253546	intergenic	1/1
CRY1-mClover3	Intron 12 of *CRY1* (5'-homology region)	Chr1: 178513956	TEX35 (intron)	1/>30
