# Supplementary material for: Live-cell imaging of circadian clock protein dynamics in CRISPR-generated knock-in cells
Source: Nat Commun. 2021 Jun 18;12:3796. doi: 10.1038/s41467-021-24086-9 (PMC8213786; doi:10.1038/s41467-021-24086-9)
Supplement: Supplementary file 3 — Description of Additional Supplementary Information [file 41467_2021_24086_MOESM3_ESM.pdf]

Description of Additional Supplementary Files

File Name: Supplementary Movie 1

Description: Animated time series of a single CRY1-mClover3 knock-in cells' nucleus over the course of 3 days after synchronization (adapted from Fig. 2a).

File Name: Supplementary Movie 2

Description: Animated time series of a single CRY1-mScarlet-I knock-in cells' nucleus over the course of 3 days after synchronization (adapted from Fig. 2a).

File Name: Supplementary Movie 3

Description: Animated time series of a single PER2-mClover3 knock-in cells' nucleus over the course of 3 days after synchronization (adapted from Fig. 2c).

File Name: Supplementary Movie 4

Description: Animated time series of a single PER2-mScarlet-I knock-in cells' nucleus over the course of 3 days after synchronization (adapted from Fig. 2c).

File Name: Supplementary Movie 5

Description: Animated time series of a single CRY1- mClover3/PER2-mScarlet-I double knock-in cells' nucleus over the course of 3 days after synchronization (adapted from Fig. 3b).
